# Structural basis of Ca^2+^-dependent activation and lipid transport by a TMEM16 scramblase

**DOI:** 10.7554/eLife.43229

**Published:** 2019-01-16

**Authors:** Maria E Falzone, Jan Rheinberger, Byoung-Cheol Lee, Thasin Peyear, Linda Sasset, Ashleigh M Raczkowski, Edward T Eng, Annarita Di Lorenzo, Olaf S Andersen, Crina M Nimigean, Alessio Accardi

**Affiliations:** 1 Department of BiochemistryWeill Cornell Medical CollegeNew YorkUnited States; 2 Department of AnesthesiologyWeill Cornell Medical CollegeNew YorkUnited States; 3 Department of Structure and Function on Neural NetworkKorea Brain Research InstituteDeaguRepublic of Korea; 4 Department of Physiology and BiophysicsWeill Cornell Medical CollegeNew YorkUnited States; 5 Department of PathologyWeill Cornell Medical CollegeNew YorkUnited States; 6 Simons Electron Microscopy CenterNew York Structural Biology CenterNew YorkUnited States; Semmelweis UniversityHungary; The University of Texas at AustinUnited States

**Keywords:** Scrambling, membrane structure, membrane channels, phospholipids, *S. cerevisiae*

## Abstract

The lipid distribution of plasma membranes of eukaryotic cells is asymmetric and phospholipid scramblases disrupt this asymmetry by mediating the rapid, nonselective transport of lipids down their concentration gradients. As a result, phosphatidylserine is exposed to the outer leaflet of membrane, an important step in extracellular signaling networks controlling processes such as apoptosis, blood coagulation, membrane fusion and repair. Several TMEM16 family members have been identified as Ca^2+^-activated scramblases, but the mechanisms underlying their Ca^2+^-dependent gating and their effects on the surrounding lipid bilayer remain poorly understood. Here, we describe three high-resolution cryo-electron microscopy structures of a fungal scramblase from *Aspergillus fumigatus*, afTMEM16, reconstituted in lipid nanodiscs. These structures reveal that Ca^2+^-dependent activation of the scramblase entails global rearrangement of the transmembrane and cytosolic domains. These structures, together with functional experiments, suggest that activation of the protein thins the membrane near the transport pathway to facilitate rapid transbilayer lipid movement.

## Introduction

The plasma membranes of eukaryotic cells are organized in an asymmetric manner; at rest, polar and charged lipids are sequestered to the inner leaflet by the activity of ATP-driven pumps. Activation of a specialized class of membrane proteins – phospholipid scramblases – causes rapid collapse of this asymmetry and externalization of negatively charged phosphatidylserine molecules. As a result, extracellular signaling networks, controlling processes such as apoptosis, blood coagulation, membrane fusion and repair, are activated (Pomorski and Menon, 2006; Bevers and Williamson, 2016; Nagata et al., 2016). The TMEM16 family of membrane proteins includes phospholipid scramblases and Cl^-^ channels (Falzone et al., 2018), all of which are Ca^2+^-dependent. Notably, TMEM16 scramblases also mediate Ca^2+^-dependent ion transport (Malvezzi et al., 2013; Scudieri et al., 2015; Yu et al., 2015; Lee et al., 2016; Lee et al., 2018). Prior structural and functional analyses of the fungal nhTMEM16 scramblase from *Nectria haematococca* identified a membrane-exposed hydrophilic groove that serves as the translocation pathway for ions and lipids (Brunner et al., 2014; Yu et al., 2015; Bethel and Grabe, 2016; Jiang et al., 2017; Lee et al., 2018). In the TMEM16A channel, this pathway is sealed from the membrane, preventing lipid access while allowing only ion permeation (Dang et al., 2017; Paulino et al., 2017a; Paulino et al., 2017b).

Phospholipid scramblases are unusual membrane proteins in the sense that their environment serves as their substrate. Activation of scramblases results in the fast and passive transbilayer movement of lipids. Thus, scramblases should affect the surrounding membrane to create a conduit between leaflets though which lipids can diffuse. Current models of lipid translocation, based on the structure of the Ca^2+^-bound conformation of the nhTMEM16 scramblase, postulate a mechanism in which lipid headgroups move through the hydrophilic permeation pathway while the tails remain embedded in the membrane core (Pomorski and Menon, 2006; Brunner et al., 2014). However, little is known about whether or how lipids and Ca^2+^ binding –the physiologic activation trigger– affect the structure of the TMEM16 scramblases. Further, it is not known how these proteins affect the surrounding membrane to enable lipid scrambling. Here, we use cryo-electron microscopy (cryo-EM) and functional experiments to address these questions. We determine the structures of a functionally characterized TMEM16 scramblase, afTMEM16 from *Aspergillus fumigatus*, in lipid nanodiscs in an inactive (Ca^2+^-free) and an active (Ca^2+^-bound) conformation. These structures allow us to define key conformational rearrangements that underlie Ca^2+^-dependent scramblase activation. Additionally, we show that scrambling is inhibited by the lipid C24:0 ceramide (Cer24:0) and determine the 3.6 Å resolution structure of the Ca^2+^-bound afTMEM16/Cer24:0-nanodisc complex. These structures, together with functional experiments, suggest that the Ca^2+^-dependent conformational rearrangements described here allow the scramblase to locally thin the membrane at the opened lipid pathway to facilitate lipid transport.

## Results

### Structure of the afTMEM16 scramblase in a lipid nanodisc

**Figure 1.**
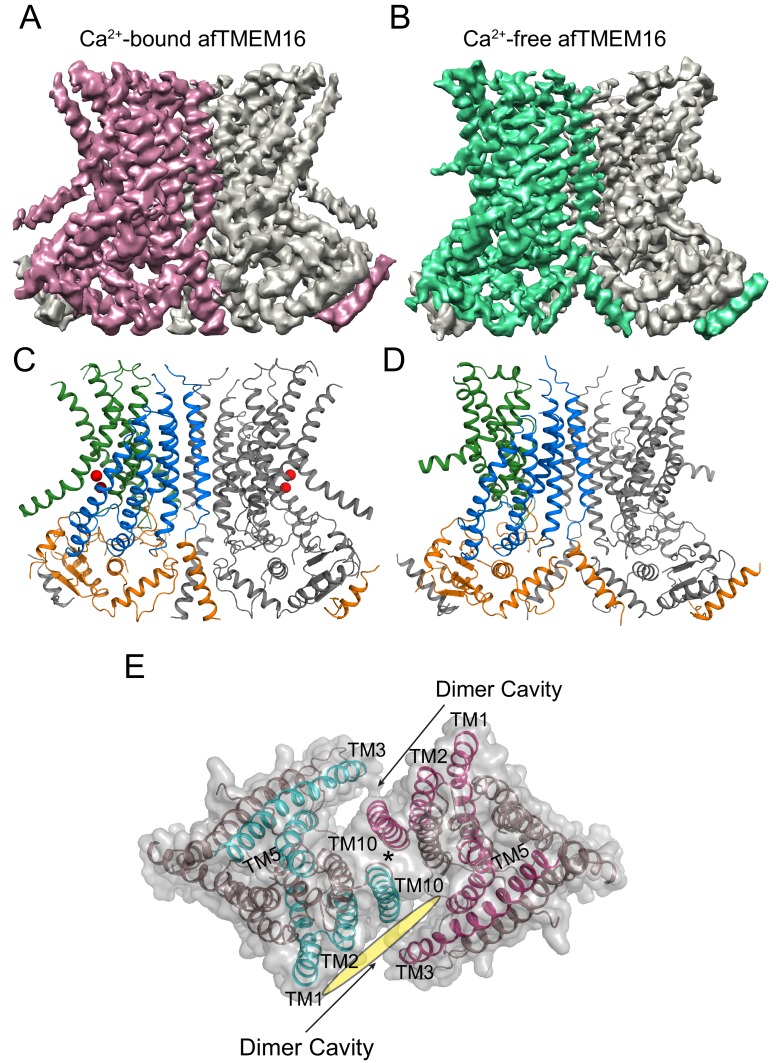
Structures of afTMEM16 in the presence and absence of Ca^2+^. (**A–B**) Masked cryo EM density maps of afTMEM16 in the presence of 0.5 mM Ca^2+^ (**A**) or in Ca^2+^-free (**B**) conditions. For clarity, one monomer is shown in gray while the other is colored in red (**A**, 0.5 mM Ca^2+^) or green (**B**, 0 Ca^2+^). (**C–D**) atomic models of afTMEM16 reconstituted in nanodiscs in the presence of 0.5 mM Ca^2+^ (**C**) and in the absence of Ca^2+^ (**D**). For clarity one monomer is gray, in the other the cytosolic domain is orange, the permeation pathway is green and the remainder of the protein is blue. Ca^2+^ ions are shown as red spheres. (**D**) Top view of Ca^2+^-bound afTMEM16 shown as maroon ribbon inside its surface representation. The dimer cavities are labeled and one of the two is highlighted with a yellow oval. * denotes the protein’s twofold axis of symmetry between the two TM10’s. To illustrate the antiparallel orientation of the two dimer cavities, the cavity-lining helices (TM1, 2, 3, 5 and 10) from the two monomers are colored in cyan and red.

To isolate conformations of afTMEM16 relevant to its scramblase activity, nanodiscs were formed from a 3:1 mixture of POPE:POPG lipids where afTMEM16 mediates lipid scrambling while its non-selective ion transport activity is silenced (Malvezzi et al., 2013; Lee et al., 2016). We used single-particle cryo-EM to determine the structures of nanodisc-incorporated afTMEM16 in the presence and absence of Ca^2+^ to resolutions of 4.0 and 3.9 Å, respectively (Figure 1). In both conditions, afTMEM16 adopts the TMEM16 fold (Figure 1; Figure 1—figure supplement 1–6), where each monomer in the dimeric protein comprises a cytosolic domain organized into a 6 α-helix (α1-α6)/3 β-strand (β1-β3) ferredoxin fold and a transmembrane region encompassing 10 α-helices (TM1-TM10) (Figure 1—figure supplement 5) (Brunner et al., 2014; Dang et al., 2017; Paulino et al., 2017a; Bushell et al., 2018). The two monomers are related by a twofold axis of symmetry at the dimer interface, formed by TM10 and the cytosolic domain (Figure 1E). The helices near the dimer interface delimit two large hydrophobic cavities, called dimer cavities, which are lined by TM1, TM2 and TM10 from one monomer and TM3, TM5 from the other (Figure 1E). In both maps, the C-terminal portion of TM6 and the linker connecting it to the short cytosolic α4 helix are not well-resolved, likely reflecting their mobility within the membrane (Figure 1A,B). When processing the data without imposing symmetry between subunits, the two monomers differ in the well-resolved density of the ~22 C-terminal residues of TM6, likely reflecting the asymmetric orientation of the protein within the nanodisc (Figure 1—figure supplement 2). The maps generated by signal subtracting the nanodisc and imposing C2 symmetry are of higher resolution and nearly identical to the non-symmetrized (C1) maps, apart from the resolved portions of TM6 (Figure 1—figure supplements 2 and 3), and were therefore used for model building.

**Figure 1—figure supplement 1.**
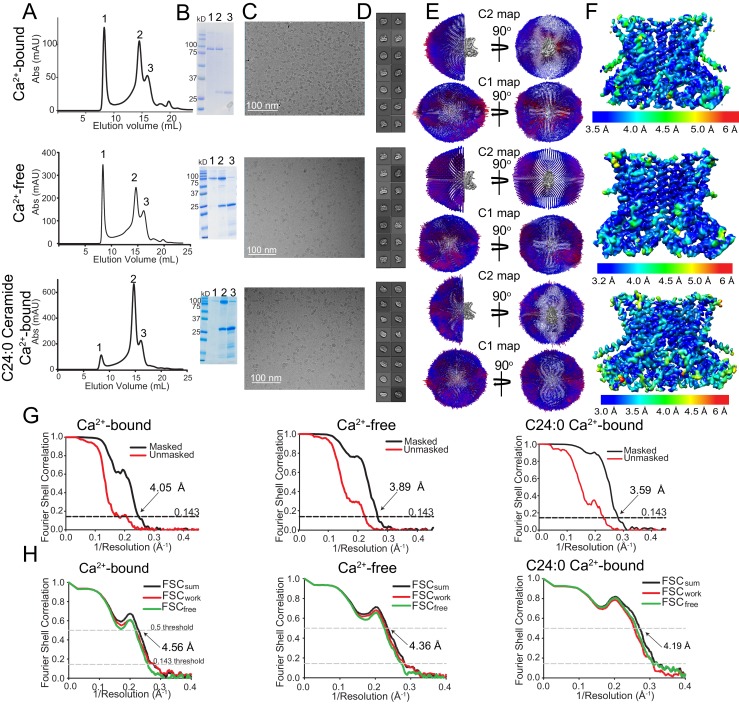
Cryo-EM characterization of afTMEM16/nanodisc complexes. (**A–F**) *Top:*+0.5 mM Ca^2+^. *Middle*: 0 Ca^2+^. *Bottom*:+0.5 mM Ca^2+^ and + 5 mole% C24:0. (**A**) Size exclusion profile of afTMEM16 in MSP1E3 nanodiscs. (**B**) SDS gel of the indicated size exclusion peaks using precision plus kaleidoscope ladder. Molecular weight (MW) of afTMEM16 is 84.5 kD and MW of MSP1E3 is 32.5 kD. (**C**) Representative cryo-EM micrographs of vitrified afTMEM16/nanodisc complexes. (**D**) Representative 2D-class averages, box size 275 Å. (**E**) Angular distribution representation of final, signal-subtracted C2 or C1 maps, number of views at each angular orientation is represented by length and color of cylinders where red indicates more views. (**F**) Final masked reconstruction colored by local resolution calculated using the Bsoft program BlocRes (Heymann, 2001; Cardone et al., 2013). (**G**) FSC plot indicating the resolution at the 0.143 threshold of final masked (black) and unmasked (red) map of afTMEM16 in the presence of 0.5 mM Ca^2+^ (left) absence of Ca^2+^ (middle), and presence of 0.5 mM Ca^2+^ and five mole% C24:0 (right). (**H**) FSC curves of refined models versus maps of afTMEM16 reconstituted in nanodiscs in the presence of 0.5 mM Ca^2+^ (left), in the absence of Ca^2+^ (middle) or with 0.5 mM Ca^2+^ and five mole% C24:0 ceramide (right). Black traces: FSC curves for the refined model compared to the final masked reconstruction (FSC_sum_). Red traces: FSC curves for the modified, refined model compared to the masked half-map 1 (FSC_work_, used during validation refinement). Green traces: FSC curves for the modified, refined model compared to the masked half- map 2 (FSC_free_, not used during validation refinement). Dashed lines show FSC threshold used for FSC_sum_ of 0.5 and for FSC_free/work_ of 0.143. Statistics for the EM analysis and model building are reported in Supplementary file 3.

**Figure 1—figure supplement 2.**
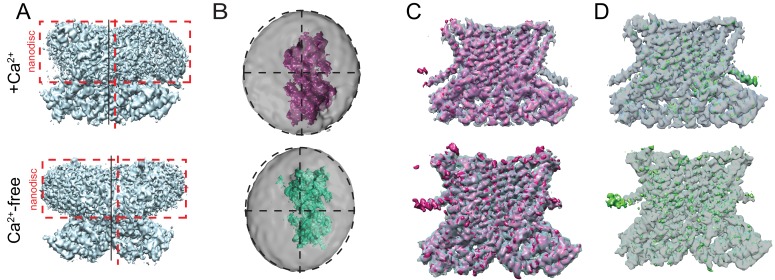
Asymmetry of afTMEM16-nanodisc complex. (**A**) Unmasked final C1 map highlighting the non-centered positioning of the protein within the nanodisc in the presence (top) and absence (bottom) of Ca^2+^. Dashed red box is drawn around the nanodisc and dashed vertical red line shows the symmetry axis of the nanodisc. Vertical black line shows the symmetry axis of the protein. (**B**) Top views of the afTMEM16-nanodisc complexes for afTMEM16 +Ca^2+^ (top, pink) and afTMEM16 Ca^2+^-free (bottom, green). The masked final maps are shown inside unmasked maps, which were low pass filtered to 10 Å and are shown at σ = 0.25. Dashed circles indicate the oval outline of nanodisc and the dashed lines denote the axis of the oval to highlight the different position of the protein map relative to the nanodiscs. (**C**) Overlay of C1 (solid pink) and C2 (transparent gray) maps for the + Ca^2+^ (top) and 0 Ca^2+^ complexes (bottom). (**D**) The difference map between the C1 and C2 maps (green) is shown inside the C2 map (gray transparent).

**Figure 1—figure supplement 3.**
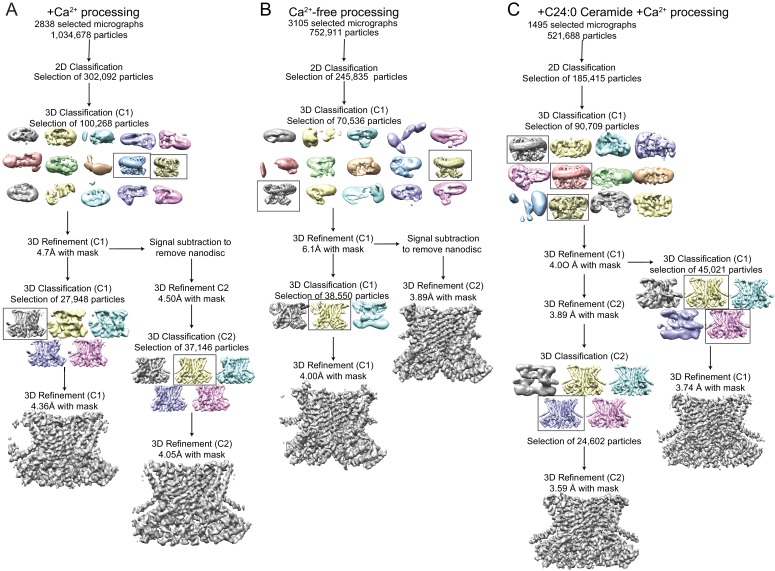
Cryo-EM data processing procedure for afTMEM16/nanodisc complexes. (**A–C**) Data processing flow chart for +Ca^2+^ (**A**), Ca^2+^-free (**B**), and +C24:0 Ceramide +Ca^2+^ (**C**). Particles picked from manually inspected micrographs were sorted with 2D and 3D classification in C1 before particle polishing and final masked refinement. For the +Ca^2+^ and Ca^2+^-free structures, particles were further classified and refined in C2 following signal subtraction of the nanodisc density. See Materials and methods for detailed procedure.

**Figure 1—figure supplement 4.**
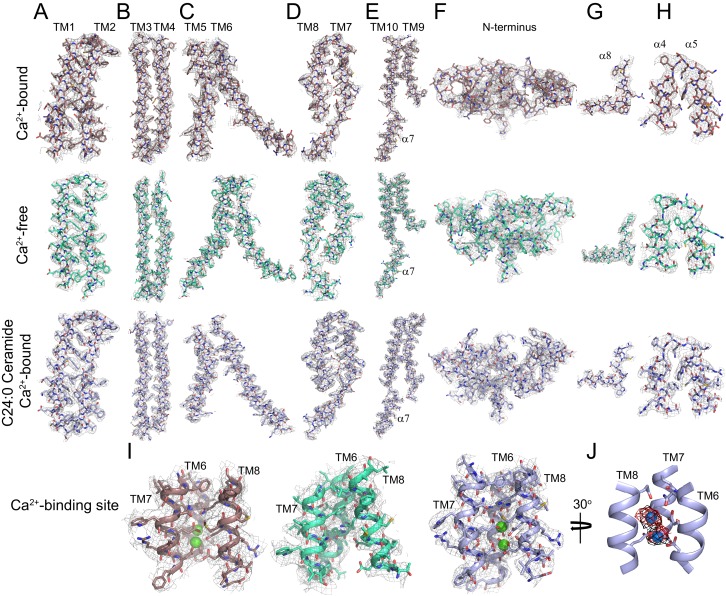
Representative cryo-EM density for afTMEM16 in 0.5 mM Ca^2+^, in 0 mM Ca^2+^ and in the presence of 0.5 mM Ca^2+^ and 5 mol% C24:0 Ceramide. (**A–I**) The cryo-EM density (gray mesh) is overlaid with the respective atomic models (sticks), Ca^2+^-bound afTMEM16 is in maroon (top panels), Ca^2+^-free is in cyan (middle panels) and Ca^2+^-bound afTMEM16 in the presence of C24:0 Ceramide is in light blue (bottom panels). Heteroatoms are colored as follows: oxygen is red, nitrogen is blue, and sulfur is yellow. (**A–E**) Transmembrane domains TM1-10 and cytosolic α7. (**F**) N-terminus containing α1–3, α6 and β1–3. (**G**) Close up of the domain-swapped region α8. (**H**) Close up of the cytosolic helices α4–5. (**I**) Ca^2+^ binding site consisting of portions of TM6-8 with Ca^2+^ ions shown in green. (**J**) Placement of Ca^2+^ ions (green) in the +Ca^2+^ + C24:0 Ceramide structure inside EM density map (black) and omit difference map (red).

**Figure 1—figure supplement 5.**
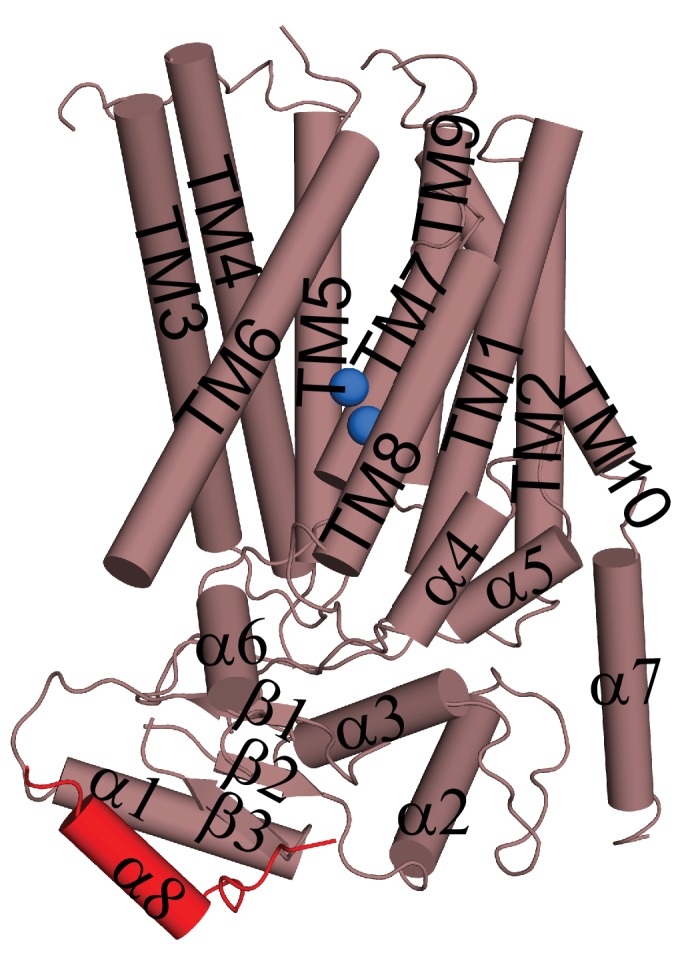
Helical organization of afTMEM16. Helices are shown as cylinders and the domain-swapped α8 from the other monomer is shown in red. Ca^2+^ ions are shown as blue spheres.

**Figure 1—figure supplement 6.**
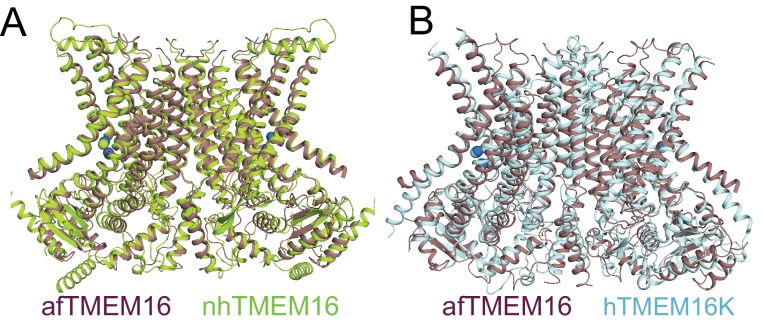
afTMEM16 in nanodiscs is very similar to nhTMEM16 and human TMEM16K in detergent. (**A–B**) Structural alignment of the atomic models of Ca^2+^-bound afTMEM16 in nanodiscs (purple) and nhTMEM16 in detergent (limon, PDBID 4WIS, Cα RMSD 1.92 Å) of hTMEM16K in detergent (light blue, PDBID 5OC9, Cα RMSD 3.69 Å).

In the presence of the functionally saturating concentration of 0.5 mM Ca^2+^ (Malvezzi et al., 2013), afTMEM16 adopts a conformation similar to that of Ca^2+^-bound nhTMEM16 and hTMEM16K in detergent (Brunner et al., 2014; Bushell et al., 2018) (Figure 1—figure supplement 5). The TM3-6 from each monomer form a peripheral hydrophilic cavity that opens to the membrane with a minimum diameter of ~5 Å. This pathway was proposed to allow entry and translocation of phospholipid headgroups (Brunner et al., 2014; Yu et al., 2015; Lee et al., 2016; Jiang et al., 2017; Lee et al., 2018; Malvezzi et al., 2018) and is analogous to the ion pathway in the TMEM16A channel (Lim et al., 2016; Dang et al., 2017; Paulino et al., 2017a; Paulino et al., 2017b; Peters et al., 2018). Thus, the presence of a lipid environment does not affect the Ca^2+^-bound conformation adopted by the scramblase.

### Ca^2+^ binding induces global rearrangements in the afTMEM16 scramblase

**Figure 2.**
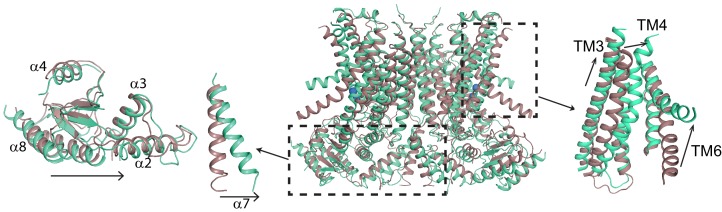
Ca^2+^-induced changes in afTMEM16. Structural alignment of afTMEM16 in the presence of 0.5 mM Ca^2+^ (maroon) and absence of Ca^2+^ (cyan) (blue sphere). Left: conformational changes in the cytosolic domain, Right: conformational changes in the lipid permeation pathway. Arrows indicate direction of movement from the Ca^2+^-bound to the Ca^2+^-free conformations.

**Figure 3.**
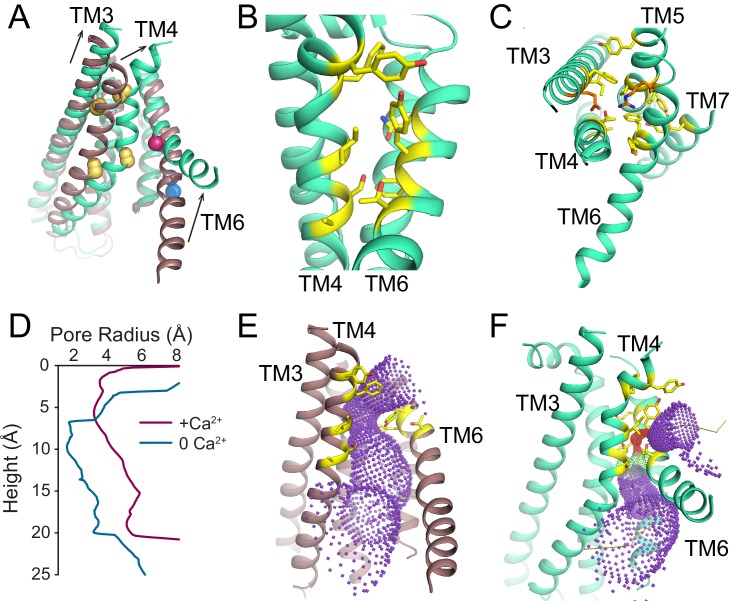
Ca^2+^-dependent rearrangements of the lipid permeation pathway. (**A**) Structural alignment of the lipid pathway with afTMEM16 in the presence (maroon) or absence (cyan) of 0.5 mM Ca^2+^. The color scheme is the same throughout the figure. Arrows indicate direction of movement from the Ca^2+^-free to the Ca^2+^-bound conformations. The lipid permeation pathway is constricted by rearrangements of TM4 around P324 and P333 (shown as yellow spheres in both structures) and TM6 at A437 (shown as a red sphere in both structures). (**B**) Close-up view of the closed permeation pathway, residues at the interface with the membrane are shown as yellow sticks. (**C**) Top view of the permeation pathway in the absence of Ca^2+^. Residues pointing inside the pathway are shown as yellow sticks. The interacting charged pair E305 and R425 are shown as orange sticks. (**D**) Diameter of the afTMEM16 lipid pathway in the presence (maroon) and absence (cyan) of Ca^2+^. The diameter was estimated using the HOLE program (Smart et al., 1996). (**E–F**) Accessibility of the lipid permeation pathway estimated using the program HOLE in the presence (**E**) or absence (**F**) of Ca^2+^. Purple denotes areas of diameter d > 5.5 Å, yellow areas where 5.5 < d < 2.75 Å and red areas with d < 2.75 Å.

To understand the changes that occur upon Ca^2+^ activation, we compared our Ca^2+^-bound and Ca^2+^-free structures of afTMEM16 and identified global conformational rearrangements of the lipid pathway, of the cytosolic domains and of the Ca^2+^ binding site (Figure 2, Video 1). In the absence of Ca^2+^, the cytosolic domains of afTMEM16 translate ~3 Å parallel to the plane of the membrane, away from the twofold axis of symmetry, such that the overall cytosolic domain becomes more expanded and the cytosolic α7 helices tilt toward the axis of symmetry with no noticeable movement of the transmembrane dimer interface (Figure 2, left panel; Figure 2—figure supplement 1). In the Ca^2+^-free conformation, the afTMEM16 lipid pathway is closed to the membrane by a pinching motion of the extracellular portions of TM4 and TM6, which move toward each other by ~7 and~3 Å, respectively (Figure 2, right panel). From the Ca^2+^-bound conformation, TM4 bends around two prolines (P324 and P333) and TM3 slides by ~6 Å to reach the Ca^2+^-free conformation (Figure 3A). Additionally, the intracellular portion of TM6 kinks around A437 by ~20^o^, inducing a ~ 16 Å vertical displacement of its terminal end (Figure 3A, Video 2). These rearrangements lead to tighter packing of side chains from TM4 and TM6 and result in exposure of a hydrophobic surface to the membrane core (Figure 3B). The pathway is also closed to ion entry by multiple stacked aromatic and hydrophobic side chains from TM3-7 (Figure 3C). The narrowest access point of the lipid pathway constricts from ~5 to ~1 Å in the absence of Ca^2+^ preventing lipid entry (Figure 3D–F). Notably, mutating residues at the interface between helices that rearrange upon Ca^2+^ binding results in severely impaired scrambling activity in the closely related nhTMEM16 homologue (Jiang et al., 2017; Lee et al., 2018) (Figure 3—figure supplement 1), highlighting the importance of these dynamic rearrangements. The global Ca^2+^-dependent rearrangements of the afTMEM16 scramblase contrast to the local conformational changes seen in the TMEM16A channel, where only TM6 bends upon Ca^2+^ions leaving the binding sites (Figure 3—figure supplement 2) (Paulino et al., 2017a).

**Figure 2—figure supplement 1.**
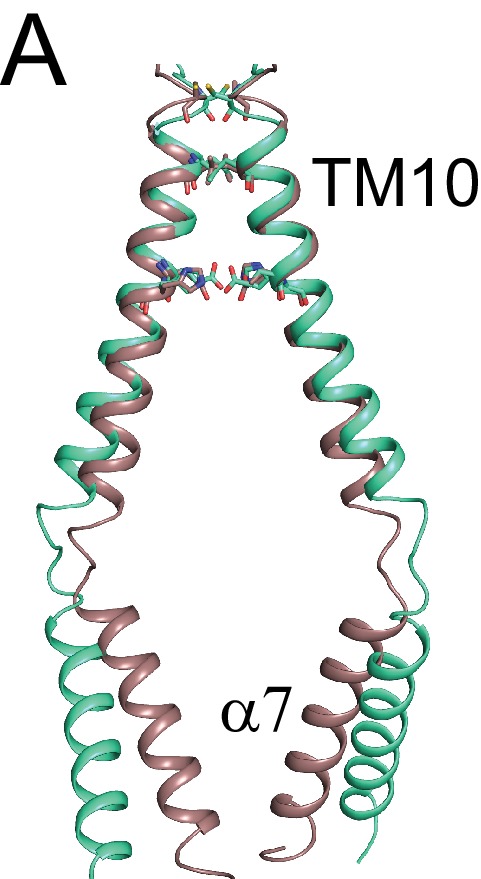
afTMEM16 dimer interface. (**A**) Structural alignment of the TM10-α7 dimer interface of afTMEM16 in Ca^2+^-bound (maroon) and Ca^2+^-free (cyan) conformations.

**Figure 3—figure supplement 1.**
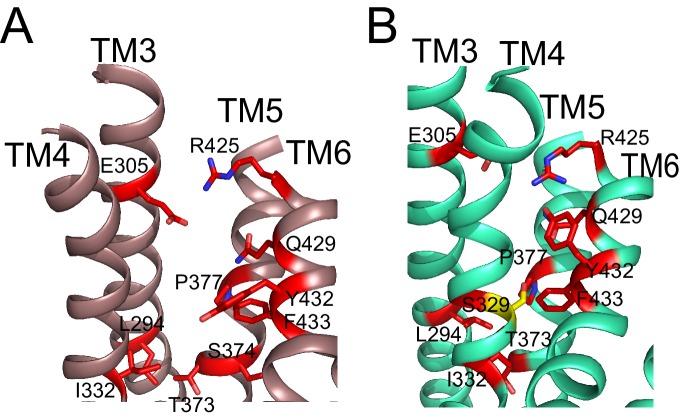
Residues important for scrambling are mapped onto afTMEM16 open and closed permeation pathways. (**A–B**) The position of recently identified residues important for lipid scrambling by nhTMEM16 (red sticks) (Jiang et al., 2017; Lee et al., 2018) are mapped onto the afTMEM16 Ca^2+^-bound (**A**) and Ca^2+^-free (**B**) structures. Mutation of theses residues reduces the rate of scrambling by >100 fold. Critical positions cluster at the rearranging interfaces between helices TM3-6. Of note is V337 (S329 in afTMEM16, shown in yellow) whose mutation to tryptophan increases scrambling activity in the absence of Ca^2+^. In the Ca^2+^-free structure, this residue is positioned at the TM4-TM6 contact point, suggesting that this substitution might prevent closure of the pathway.

**Figure 3—figure supplement 2.**
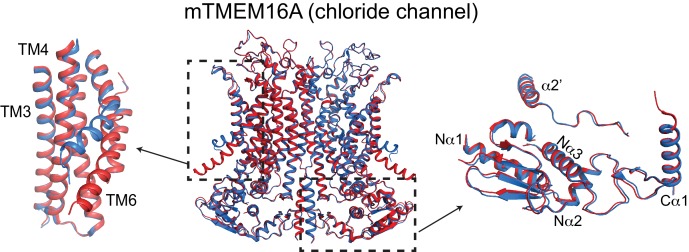
Ca^2+^-induced changes in TMEM16A. Structural alignment of TMEM16A in the presence of 0.5 mM Ca^2+^ (PDBID: 5OYB, red) and absence of Ca^2+^ (PDBID: 5OYG, blue). Left: conformational changes in ion permeation pathway. Right: view of cytosolic domains showing a lack of movement upon Ca^2+^-binding.

**Video 1.** Ca^2+^-activation of afTMEM16. Morph between Ca^2+^-free and Ca^2+^-bound conformations of afTMEM16. For clarity one monomer is shown in gray. In the other, the cytosolic domain is in orange, the lipid permeation pathway (TM3-7) in green, and the rest of the protein in blue (TM1-2 and TM8-10). Note the downward motion of TM6 and movement of the cytosolic domains parallel to the membrane plane.

**Video 2.** Ca^2+^-dependent movement of the afTMEM16 lipid pathway. Close-up view of the rearrangements undergone by the afTMEM16 lipid pathway (TM3-7) upon Ca^2+^ binding. Note that upon Ca^2+^ binding TM4 straightens and moves out of the pathway, TM3 slides downward and TM6 moves out of the pathway and bends downwards to open the pathway.

### Ca^2+^-dependent conformational changes of the regulatory Ca^2+^-binding sites

**Figure 4.**
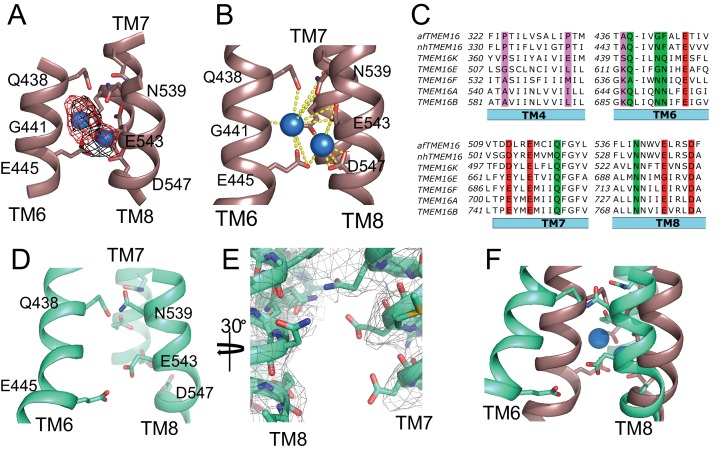
afTMEM16 Ca^2+^-binding site and conformational changes. (**A**) Close up view of the Ca^2+^-binding site, with key coordinating residues shown as sticks. The density corresponding to the Ca^2+^ ions (blue spheres) from the experimental map is shown in black and the density from the calculated omit difference map is shown in red. The peak density corresponding to the Ca^2+^ ions in the omit difference maps (red mesh) is σ = 13 and 7. (**B**) Ca^2+^ coordination in afTMEM16. (**C**) Structure-based sequence alignment of the Ca^2+^-binding site and gating region of TMEM16 proteins. The alignment was generated using PROMALS3D (Pei and Grishin, 2014). Highlighted residues: conserved acidic (red) or polar (green) side chain in Ca^2+^ binding site, and the residues around which TM4 and TM6 bend (pink). (**D**) Close-up of the Ca^2+^-binding site in the absence of ligand. Coordinating residues on TM6 and TM8 are labeled. (**E**) EM density of residues lining the Ca^2+^-binding site in the absence of Ca^2+^ highlighting the lack of density for Ca^2+^ ions. (**F**) Structural alignment of the binding site with and without Ca^2+^.

The transmembrane region of afTMEM16 contains two Ca^2+^-binding sites, located between TM6, TM7, and TM8 (Figure 4A,B). In the presence of 0.5 mM Ca^2+^, both sites are occupied by calcium ions, as evidenced by the strong densities in the cryo-EM map and in an ‘omit’ difference map, calculated between experimental data and simulated maps not containing Ca^2+^ (Figure 4A). The bound Ca^2+^ ions are coordinated by five conserved acidic residues (E445 on TM6, D511 and E514 on TM7, and E534 and D547 on TM8), three polar residues (Q438, Q518, N539), and the unpaired main chain carbonyl of G441 (Figure 4A–C). This coordination is similar to that seen in the nhTMEM16 and hTMEM16K scramblases as well as in the TMEM16A channel, consistent with the evolutionary conservation of the Ca^2+^-binding residues (Figure 4C) (Yu et al., 2012; Malvezzi et al., 2013; Terashima et al., 2013; Brunner et al., 2014; Tien et al., 2014; Lim et al., 2016; Dang et al., 2017; Paulino et al., 2017a; Bushell et al., 2018). In the absence of Ca^2+^, the binding site is disrupted by the movement of TM6 which displaces the three residues participating in the site (Q438, G441 and E445) (Figure 4D–F, Video 3). Additional rearrangements of TM8, displacing N539 and E543, further disrupt the Ca^2+^-binding site (Figure 4D–F, Video 3). No Ca^2+^ density was visible in the cryo-EM map (Figure 4E), confirming that the scramblase is in a Ca^2+^-free conformation. The movement of TM6 and TM8 in opposite directions partially relieves the electrostatic repulsion of the uncoordinated acidic side chains that form the Ca^2+^-binding site and opens a wide, water-accessible, conduit for ions to access the binding site from the intracellular solution.

**Video 3.** Ligand-dependent rearrangements of the Ca^2+^binding site. Close-up view of the rearrangements undergone by the afTMEM16 ligand binding site upon Ca^2+^ binding. TM6 and TM7 are shown in green and TM8 is shown in blue. The residues directly involved in Ca^2+^ coordination are shown as sticks. Upon Ca^2+^ binding, TM6 moves toward TM7 and TM8 while TM8 tilts back toward TM7 to form the binding site.

### Structural analysis of the afTMEM16/nanodisc complex

**Figure 5.**
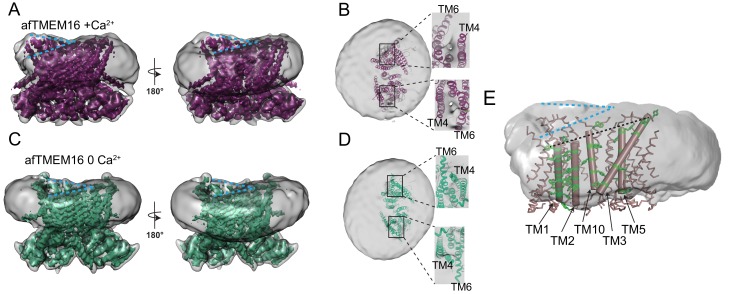
afTMEM16 affects the surrounding membrane. (**A and C**) The unmasked maps (gray) of the afTMEM16/nanodisc complex in the presence (**A**) and absence of Ca^2+^ (**C**) are shown low pass filtered to 10 Å at σ = 0.4 with front (left panels) and back (right panels) views. The masked map of the afTMEM16 scramblase in 0.5 mM Ca^2+^ (**A**), in purple) or in 0 Ca^2+^ (**C**), in green) are shown inside the respective unmasked maps. Blue dashed lines highlight the bending at the dimer cavities. (**B and D**) Top view of the unmasked map (grey) with (**B**) and without (**D**) Ca^2+^ low-pass filtered to 10 Å at σ = 0.1 with the respective atomic models shown as ribbons inside. Insets show close-up views of the nanodiscs density at the permeation pathways. Note that the insets are displayed at a tilted angle compared to the top views to visualize the lipid pathway. (**E**) Bending of the outer leaflet at the dimer cavities in the presence of 0.5 mM Ca^2+^. The low-pass filtered map of the complex (from **A**) was segmented and the isolated nanodisc density is shown. The afTMEM16 transmembrane region is shown in ribbon with the helices lining one dimer cavity (TM1, 2, 3, 5, and 10) in cartoon representation. Aromatic residues are shown as green sticks. Blue dashed lines trace the upper membrane leaflet at the two sides of the lipid permeation pathway highlighting the opposite slant. Black dashed line traces the slope of the helices lining the dimer cavity.

Because these structures are of protein/nanodisc complexes, they also provide insights into the Ca^2+^-dependent interactions between the scramblase and the nanodisc membrane (Figure 5). Inspection of these maps reveals that the nanodiscs containing afTMEM16 are bent along the two dimer cavities of the scramblase (Figure 5A,C) and that there is a region of low density at the open lipid pathway (Figure 5B). The nanodisc density is a convolution of the lipid molecules and the MSP1E3 protein that defines the nanodisc boundary, suggesting that presence of the afTMEM16 scramblase affects the orientation of both.The bending along the dimer cavities of afTMEM16 is seen in the 3D reconstructions from all datasets presented here, suggesting that it does not depend on the presence of Ca^2+^, on the conformation of the protein or processing algorithm (Figure 5A,C, Figure 5—figure supplement 1–3, Supplementary file 1). Remarkably, bending of the nanodisc is also visible in 2D classes of particles containing afTMEM16, but is absent from protein-free ones (Figure 5—figure supplement 4). In all maps, the membrane appears to be thicker around the long TM3 helix in one monomer and thinner at the short TM1 of the other (Figure 5E). This bending is visible along both dimer cavities, irrespective of the positioning of the scramblase within the nanodisc (Figure 5A,C, Figure 5—figure supplement 1). The independence of the observed membrane bending on Ca^2+^ is consistent with the lack of Ca^2+^-dependent rearrangements undergone by these cavities (Figure 3A). The membrane slant matches the tilted plane defined by the extracellular ends of the five, dimer cavity-lining helices (Figure 5E) suggesting that it is caused by the architecture of afTMEM16.

**Figure 5—figure supplement 1.**
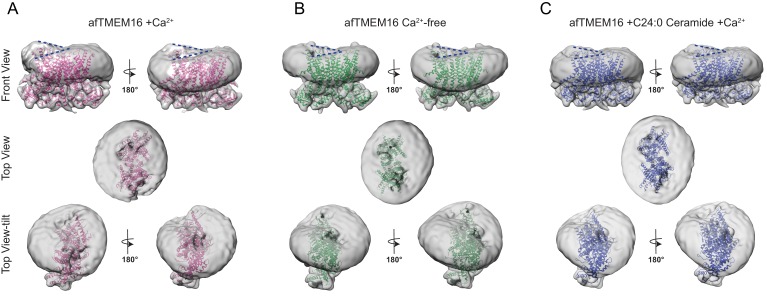
Ca^2+^-dependent and Ca^2+^-independent effects of afTMEM16 on nanodisc density. (**A–C**) The unmasked maps of the afTMEM16/nanodisc complex in the presence of 0.5 mM Ca^2+^ (**A**), in Ca^2+^-free conditions (**B**) and with 0.5 mM Ca^2+^ and 5 mol% C24:0 Ceramide (**C**) are shown low-pass filtered to 10 Å and at σ = 3.5. The respective atomic models are shown inside in pink (0.5 mM Ca^2+^, (**A**)), green (Ca^2+^-free, (**B**)) and blue (0.5 mM Ca^2+^ and 5 mol% C24:0 Ceramide, (**C**)). Top panels: front and back (rotated 180^o^) views; middle: top views; bottom: top views tilted by ~30^o^ relative to the middle panels to visualize the permeation pathway in each monomer (the two panels are rotated by 180^o^). Dashed blue lines in the top panels trace the membrane bending near the dimer cavities. Note how similar bending is observed in the front and back views.

**Figure 5—figure supplement 2.**
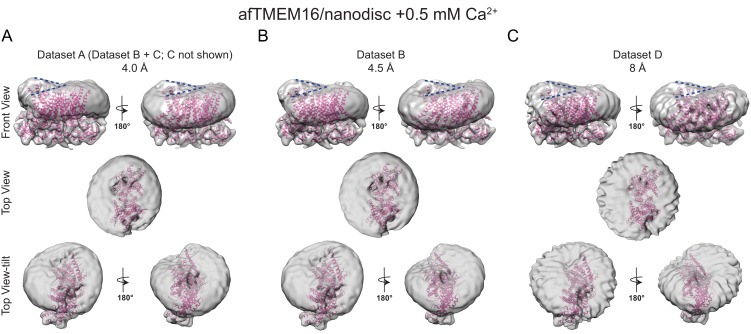
The effects of Ca^2+^-bound afTMEM16 on the surrounding membrane are seen in three independent datasets with varying resolutions. (**A–C**) The unmasked maps of afTMEM16/nanodisc complex in the presence of 0.5 mM Ca^2+^ determined from three datasets are shown low-pass filtered to 10 Å and at σ = 3.5. The high-resolution afTMEM16 +Ca^2+^atomic model (pink) is shown inside the unmasked maps. (**A**) the map derived from the 4.0 Å resolution dataset (dataset A, Supplementary file 1) (**B**) the map obtained from the processing of dataset B (Supplementary file 1) resulting in a map with a resolution of 4.5 Å; (**C**) 3D reconstruction from dataset D (Supplementary file 1) that resulted in a map with a resolution of ~8 Å. Top: front and back (rotated 180^o^) views; middle: top views; bottom: top views tilted by ~30^o^ relative to the middle panels to visualize the permeation pathway in each monomer (the two panels are rotated by 180^o^). Dashed blue lines in the top panels trace the membrane bending near the dimer cavities. Note how similar bending is observed in the front and back views.

**Figure 5—figure supplement 3.**
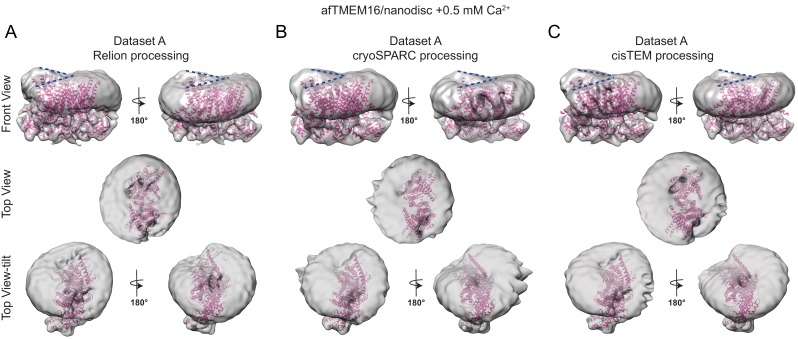
The effects of Ca^2+^-bound afTMEM16 on the surrounding membrane do not depend on the processing algorithm. (**A–C**) The unmasked maps of the afTMEM16/nanodisc complex in the presence of 0.5 mM Ca^2+^ determined from dataset A (see Supplementary file 1) are shown low-pass filtered to 10 Å and at σ = 3.5. The high-resolution afTMEM16 +Ca^2+^atomic model (pink) is shown inside the unmasked maps. The maps were obtained with processing the dataset in Relion (**A**), cryoSPARC (**B**) or cisTEM (**C**). Top: front and back (rotated 180^o^) views; middle: top views; bottom: top views tilted by ~30^o^ relative to the middle panels to visualize the permeation pathway in each monomer (the two panels are rotated by 180^o^). Dashed blue lines in the top panels trace the membrane bending near the dimer cavities. Note how similar bending is observed in the front and back views.

**Figure 5—figure supplement 4.**
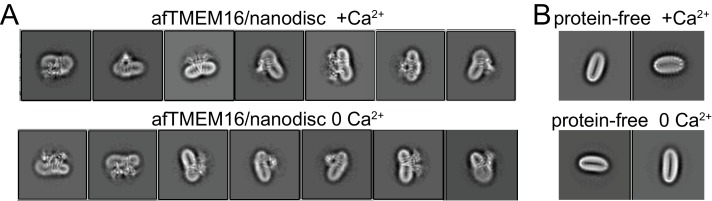
Altered membrane organization induced by activation of afTMEM16. (**A–B**) Close-up view of representative 2D classes (box size 275 Å) of Ca^2+^-bound (0.5 mM) (top) and Ca^2+^-free (bottom) nanodisc-reconstituted afTMEM16 (taken from Figure 1 —figure supplement 1D). Note how the bending of the nanodisc induced by afTMEM16 is visible in the 2D classes. (**B**) Close up view of 2D class averages (box size 275 Å) of empty (without protein) nanodiscs from the +Ca^2+^ (top) and Ca^2+^-free (bottom) data sets. Note how the empty nanodiscs are flat and not bent.

In contrast to the membrane bending, the region of weaker density of the nanodisc membrane near the lipid pathway depends on the scramblase conformation (Figure 5B,D). In the three datasets collected in the presence of 0.5 mM Ca^2+^, the density between the TM4 and TM6 helices that delimit the open lipid transport pathway is weaker than in the rest of the complex (Figure 5B, insets, Figure 5—figure supplement 2). This weakening can be seen in both subunits irrespective of the distance of the pathway from the nanodisc edge (Figure 5B, insets). In the absence of Ca^2+^, no region of weak density is visible (Figure 5D, inset) as the conformational rearrangement of TM4 and TM6 pinches shut the permeation pathway. The weaker density suggests that the membrane is thinner and/or more disordered near the open lipid pathway of an active scramblase.

### Lipid dependence of scrambling by afTMEM16

**Figure 6.**
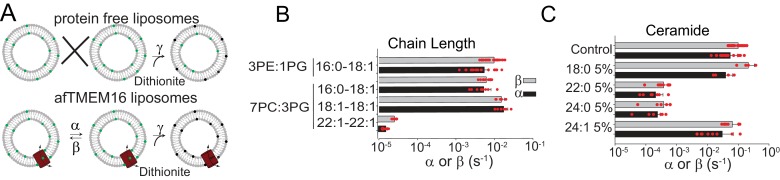
Dependence of afTMEM16 on membrane lipids. (**A**) Schematic of the in vitro scramblase assay. Liposomes are reconstituted with NBD-labeled phospholipids (green) that distribute equally in the two leaflets. Addition of extraliposomal sodium dithionite reduces the NBD fluorophore (black), causing 50% fluorescence loss in protein-free vesicles (top panel). When a scramblase is present (bottom panel), all NBD-phospholipids become exposed to dithionite, resulting in complete loss of fluorescence (Malvezzi et al., 2013). (**B–C**), Forward (α, black) and reverse (β, grey) scrambling rate constants of afTMEM16 in 0.5 mM Ca^2+^ as a function of lipid chain length (**B**) or addition of 5 mole% of different ceramides (**C**). Rate constants were determined by fitting the fluorescence decay time course to Eq. 1. For the chain length experiments liposomes were formed either from a 3 POPE: 1 POPG or a 7 POPC: 3 POPG mixture (**B**). A 3 POPE: 1 POPG mixture was used for the reconstitutions containing ceramide lipids (**C**). Data is reported as mean ±S.D. Red circles denote individual experiments. Values and exact repeat numbers are reported in Supplementary files 2,4.

The hypothesis that afTMEM16 alters the membrane to scramble lipids suggests that scrambling should be impaired by thicker membranes and by bilayer modifying lipids. To increase the membrane thickness, we reconstituted afTMEM16 into liposomes composed of lipids of varying acyl chain length and saturation. The rate of scrambling was determined with a dithionite-based fluorescence assay (Figure 6A) (Malvezzi et al., 2013; Malvezzi et al., 2018). We found that afTMEM16 is equally active in liposomes formed from mixtures of POPE:POPG or POPC:POPG lipids, which have C16:0 and C18:1 acyl chains, and in liposomes formed from DOPC:DOPG lipids, where both acyl chains are C18:1 (Figure 6B, Figure 6—figure supplement 1, Supplementary file 2). This suggests that the saturation of the acyl chains does not influence afTMEM16 scrambling activity. In contrast, increasing membrane thickness by ~7 Å by forming liposomes with DEPC:DEPG lipids (Lewis and Engelman, 1983), with C22:1 acyl chains, reduces the scrambling rate of afTMEM16 by ~500 fold in the presence of Ca^2+^ and ~20-fold in the absence of Ca^2+^ (Figure 6A–B, Figure 6—figure supplement 1, Supplementary file 2). The observation that scrambling is inhibited in thicker membranes is consistent with our hypothesis that afTMEM16 thins the membrane to scramble lipids.

**Figure 6—figure supplement 1.**
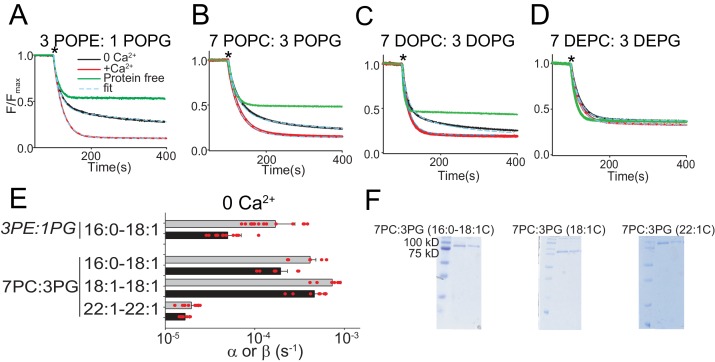
Functional modulation of lipid scrambling by acyl chain length. (**A–D**) Representative time courses of dithionite-induced fluorescence decay in protein-free liposomes (green) or in afTMEM16 proteoliposomes with (red) or without (black) Ca^2+^. Cyan dashed lines represent the fit to Eq. 1 (Malvezzi et al., 2018). * denotes addition of dithionite. Liposomes were formed from 3 POPE: 1 POPG (**A**), 7 POPC: 3 POPG (**B**), 7 DOPC: 3 DOPG (**C**), or 7 DEPC: 3 DEPG (**D**) mixtures. Please note that in 7 DEPC: 3 DEPG liposomes (**D**) the steady state value of the fluorescence decay of the protein-free liposomes is below 0.5. This reflects an asymmetric initial distribution of the NBD-PE probe rather than compromised liposome integrity, as the fluorescence reaches a plateau and remains stable for the duration of the experiment. (**E**) Forward (α, black) and reverse (β, gray) scrambling rate constants in the absence of Ca^2+^ as a function of lipid chain length. Data in panel E is reported as mean ±SD. Red circles denote individual experiments. Values and exact repeat numbers are reported in Supplementary file 3 . (**F**) SDS-PAGE gels of liposomes containing afTMEM16 throughout the reconstitution confirming the presence of the protein in the conditions without scrambling activity as compared to the associated control 22:1C PC-PG (right). Samples were taken from the reconstitution after the first biobead change (left) and after the last (right). In all gels, the final band represents the protein present in the liposomes when the experiment was completed.

**Figure 6—figure supplement 2.**
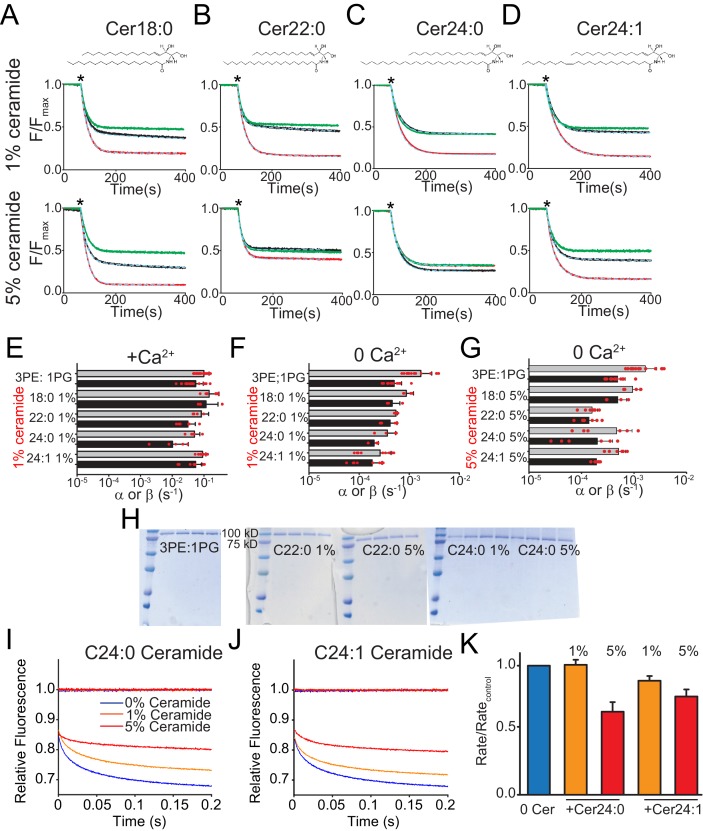
Functional modulation of lipid scrambling by addition of ceramide lipids. (**A–D**) Representative time courses of dithionite-induced fluorescence decay in control liposomes formed from a 3 POPE: 1 POPG mixtures with C18:0 Ceramide (**A**), C22:0 Ceramide (**B**), C24:0 Ceramide (**C**) or C24:1 Ceramide (**D**) at one mole% (top panels) or at five mole% (bottom panels). Please note that in the presence of 5% C24:0 Ceramide (**C**) the steady state value of the fluorescence decay of the protein-free liposomes is below 0.5. This does not reflect compromised liposome integrity, as evidenced by the fact that the fluorescence reaches a plateau and remains stable for the duration of the experiment, rather it reflects an asymmetric initial distribution of the NBD-PE probe. (**E–G**) Forward (α, black) and reverse (β, grey) scrambling rate constants of afTMEM16 with one mole% ceramides with (**E**) or without Ca^2+^ (**F**) or with 5 mole% Ceramide and without Ca^2+^ (**G**). Data in panels E-G is reported as mean ±SD. Red circles denote individual experiments. Values and exact repeat numbers are reported in Supplementary file 4. (**H**) SDS-PAGE gels of liposomes containing afTMEM16 throughout the reconstitution confirming the presence of the protein in the conditions without scrambling activity as compared to the associated control (C22:0 ceramide-middle and C24:0 ceramide-right). Samples were taken from the reconstitution after each of the four biobead changes. In all gels, the final band represents the protein present in the liposomes when the experiment was completed. (**I–J**) Representative time course of gramicidin A (gA)-mediated Tl^+^-induced quenching of ANTS in 3 POPE:1 POPG large unilamellar vesicles + C24:0 ceramide (**I**) or C24:1 ceramide (**J**). Blue trace: control –no ceramide; orange trace: 1% ceramide; red trace: 5% ceramide. (**K**) Normalized rate of the Tl^+^-induced fluorescence quenching. The quenching rate was determined by fitting a stretched exponential (Eq. 3) to the fluorescence time course in **I–J**). The quenching rate reflects the gA monomer↔dimer equilibrium that is affected by the chemicophysical properties of the membrane where a reduction in the rates reflects a stiffening and/or reduction in fluidity of the membrane.

Among naturally occurring bilayer-modifying constituents of cellular membranes, we focused our attention on ceramides, as these sphingolipids regulate cellular processes that involve activation of phospholipid scramblases, such as blood coagulation, inflammation and apoptosis (Deguchi et al., 2004; Hannun and Obeid, 2008; Borodzicz et al., 2015; Cantalupo and Di Lorenzo, 2016; Deguchi et al., 2017). We found that addition of physiological levels of long chain ceramides potently inhibits scrambling by reconstituted afTMEM16 (Figure 6C, Figure 6—figure supplement 2A–H). Among the tested ceramides, C24:0 Ceramide (Cer24:0) inhibits scrambling ~250 fold when added at 5 mole% (Figure 6C). The inhibitory effect depends on ceramide concentration, with minimal effects at 1 mole%, as well as acyl chain saturation, as Cer24:1 is nearly inert (Figure 6C, Figure 6—figure supplement 2). The length of the ceramide acyl chain is also important as Cer22:0 is as potent as Cer24:0 while Cer18:0 has minimal effect at 5 mole% (Figure 6C, Figure 6—figure supplement 2F). The long chain ceramides also inhibit scrambling in the absence of Ca^2+^ by ~10-fold (Figure 6—figure supplement 2G). The inhibitory effects of ceramides or thicker bilayers do not reflect impaired reconstitution of the protein (Figure 6—figure supplement 2H). We used a gramicidin-based fluorescence quench assay (Ingólfsson and Andersen, 2010) to investigate whether Cer24:0 and Cer24:1 differentially affect bulk membrane properties, such as thickness and fluidity. The assay monitors alterations in the gramicidin monomer↔dimer equilibrium, which varies with changes in membrane thickness and elasticity (Andersen et al., 2007; Lundbæk et al., 2010). Addition of Cer24:0 or Cer24:1 comparably reduces gramicidin activity (Figure 6—figure supplement 2I–K), indicating that both ceramides stiffen and/or thicken the membrane to a similar extent. The comparable effects of Cer24:0 and Cer24:1 on membrane properties suggest that their distinct effects on afTMEM16 scrambling might reflect specific interactions with the scramblase and/or their differential ability to form gel-like microdomains in membranes (Pinto et al., 2008; García-Arribas et al., 2017; Alonso and Goñi, 2018).

### Structure of the Ca^2+^-bound and ceramide inhibited afTMEM16/nanodisc complex

**Figure 7.**
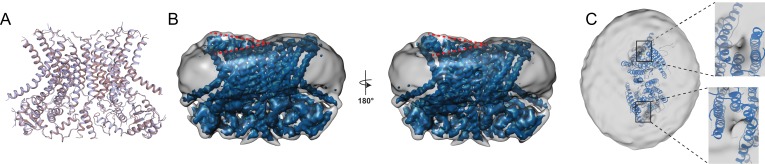
Structure of afTMEM16 in the presence of C24:0 ceramide. (**A**) Structural alignment of Ca^2+^-bound afTMEM16 with (light blue) and without C24:0 ceramide (maroon) with a Cα r.m.s.d. <1 Å. (**B**) The unmasked maps (gray) of the afTMEM16/nanodisc complex in the presence of 0.5 mM Ca^2+^ and 5 mol% C24:0 ceramide is shown low pass filtered to 10 Å at σ = 0.4 with front (left panel) and back (right panel) views. The masked map of the afTMEM16 scramblase in 0.5 mM Ca^2+^ and 5 mol% C24:0 ceramide is shown inside the respective unmasked maps. Red dashed lines highlight the membrane bending. (**C**) Top view of unmasked map (grey) low pass filtered to 10 Å at σ = 0.1 with the atomic models inside. Insets show the density at the permeation pathway. Note that the insets are displayed at a tilted angle compared to the top views to visualize the changes in density at the lipid pathway.

To understand how long-tail ceramides affect scrambling, we determined the 3.6 Å resolution structure of Ca^2+^-bound afTMEM16 in nanodiscs containing 5 mole% Cer24:0 (Figure 7; Figure 1—figure supplement 1–4, Figure 7—figure supplement 1), a concentration that drastically inhibits activity (Figure 6C). The protein adopts a conformation where the lipid pathway is open to the membrane, both Ca^2+^-binding sites are occupied, and is nearly identical to the Ca^2+^-bound active state, with an overall Cα r.m.s.d. <1 Å (Figure 7A). No individual lipids are resolved within the pathway, while several densities that we attributed to partial acyl chains are visible in the dimer cavity (Figure 7—figure supplement 1). In the Cer24:0-inhibited structure, the nanodisc membrane bends along the dimer cavities in a similar manner to what was seen in the Ca^2+^-bound and Ca^2+^-free structures, (Figure 7B). Interestingly, the density near the lipid pathway in the presence of Cer24:0 appears to be less weakened (Figure 7C) than that seen in the Ca^2+^-bound structure (Figure 5B), even though the pathway is open in both structures. While direct comparisons of the densities between the two structures are difficult because of their different resolutions, it is tempting to speculate that the density of the nanodisc membrane in this area correlates with the activity of the protein. Further work will be required to test this possibility. The finding that afTMEM16 adopts a Ca^2+^-bound conformation with open lipid pathways suggest that long chain ceramides do not inhibit scrambling by preventing the Ca^2+^-dependent opening conformational transition. Rather, our findings suggest that the physico-chemical properties of the membrane determine whether lipid scrambling actually occurs.

**Figure 7—figure supplement 1.**
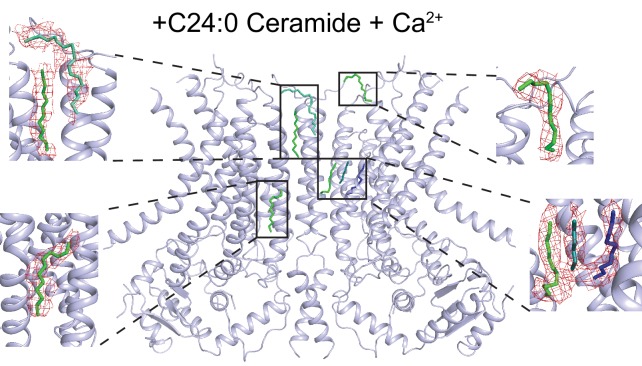
Lipid-like density in the dimer cavity. Atomic model of afTMEM16 in in the presence of 0.5 mM Ca^2+^ and 5 mole% C24:0 Ceramide with location of lipid acyl chains within the side dimer cavity. Insets show acyl chains colored by ligand ID (D12-green, D10-blue, 8K6-cyan, OCT-teal) into the difference map density (in red) between the experimental density map and the theoretical map calculated from the model with lipids omitted. Only the lipids in one dimer cavity are shown for clarity.

## Discussion

Despite recent advances (Falzone et al., 2018), the molecular mechanisms underlying the Ca^2+^-dependent activation of the TMEM16 scramblases and their interactions with the surrounding membrane lipids remain poorly understood. Here, we use cryo electron microscopy to determine the structure of a functionally well-characterized TMEM16 family member, afTMEM16 (Malvezzi et al., 2013; Lee et al., 2016; Malvezzi et al., 2018), in a membrane environment. Our results show that the afTMEM16 scramblase reconstituted in lipid nanodiscs undergoes global conformational rearrangements upon Ca^2+^ binding (Figures 1–4). These rearrangements involve the closure of the pathway via a pinching motion of TM4 and TM6, the upward motion of TM3 and the dilation of the Ca^2+^-binding site. The global nature of these rearrangements differs greatly from those seen in the TMEM16A channel, where only TM6 moves in response to Ca^2+^ binding (Dang et al., 2017; Paulino et al., 2017a) (Figure 3—figure supplement 2). The movements we observe in afTMEM16, bear striking similarities to those predicted by MD simulations for the nhTMEM16 scramblase upon removal of Ca^2+^ (Jiang et al., 2017). Notably, similar rearrangements are observed in the human TMEM16K scramblase (Bushell et al., 2018), supporting their evolutionary conservation.

**Figure 8.**
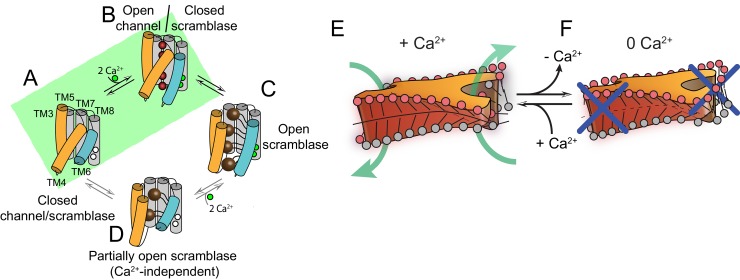
Proposed mechanisms for gating and membrane remodeling by TMEM16 scramblases. (**A–D**) Ca^2+^-dependent gating scheme for TMEM16 scramblases. The α helices lining the lipid pathway and Ca^2+^-binding site (TM3-8) are shown as cylinders. The two gating elements (TM3-4 and TM6) are colored in orange and blue, respectively. When the Ca^2+^ binding sites are empty TM4 and TM6 are bent and occlude the pathway, resulting in a closed scramblase (PDBID: 6DZ7) (**A**). Upon Ca^2+^ binding, TM6 straightens and partly disengages from TM4, giving rise to a pathway that is closed to lipids but that can potentially allow ion permeation (a mTMEM16A-like state, PDBID:5OYG) (**B**). Rearrangement of TM6 promotes the straightening of TM4, resulting in an open lipid pathway (PDBID: 4WIS, 6E0H) (**C**). Straightening of TM4 in the absence of Ca^2+^, gives rise to a partially open lipid pathway that might allow the experimentally observed Ca^2+^-independent lipid scrambling (Malvezzi et al., 2013) (**D**). If the straightening of TM4 is energetically unfavorable, the protein is restricted to visiting only states (**A**) and (**B**), green shaded area. This would give rise to the observed Ca^2+^-dependent gating behavior of the TMEM16 channels. (**E–F**) proposed mechanism for membrane remodeling by the afTMEM16 dimer. The slanted architecture of the dimer cavity primes the membrane by bending it in opposite directions on the two sides of the lipid translocation pathway. In the presence of Ca^2+^ the pathway is open, enabling the formation of a membrane area that is thin and forming a conduit through which lipid headgroups can translocate between the two leaflets (**E**). In the absence of Ca^2+^ the pathway is closed preventing lipid scrambling (**F**).

Our structural and functional experiments provide detailed insights into the activation mechanism of TMEM16 phospholipid scramblases (Figures 1–4) and how these proteins alter the surrounding membrane to facilitate the transfer of lipids between leaflets (Figures 5–7). The Ca^2+^-free and Ca^2+^-bound structures of afTMEM16 define the extremes of the Ca^2+^-dependent activation process and suggest that opening of the lipid pathway is primarily controlled by two structural elements, TM4 and TM6 (Figure 8). Without Ca^2+^, TM4 and TM6 are bent, sealing the pathway from the lipid membrane (Figure 8A). The first step in activation is presumably Ca^2+^ binding, which facilitates the transition of TM6 to a straight conformation and its disengagement from TM4, allowing TM6 to move toward TM8 and complete the formation of the Ca^2+^-binding site (Figure 8B). The resulting proposed conformation is similar to that of Ca^2+^-bound TMEM16A, where TM6 is straight but lipid access is prevented by a bent TM4 (Dang et al., 2017; Paulino et al., 2017a). Notably, an intermediate, Ca^2+^-bound conformation with a closed lipid pathway has been recently observed for the hTMEM16K scramblase (Bushell et al., 2018). Straightening of the TM4 helix opens the lipid pathway to enable lipid translocation, as seen in Ca^2+^-bound afTMEM16 and nhTMEM16 (Figure 8C)(Brunner et al., 2014). To complete the gating scheme, we propose a state where TM4 is straight and TM6 is bent (Figure 8D), which would give rise to a partially opened lipid pathway. This conformation, while not yet observed experimentally, would account for the low, basal activity of reconstituted scramblases in the absence of Ca^2+^ (Figure 6—figure supplements 1 and 2) (Malvezzi et al., 2013; Brunner et al., 2014; Lee et al., 2016; Lee et al., 2018; Malvezzi et al., 2018). Within this gating mechanism, Ca^2+^-activated TMEM16 channels would naturally arise from scramblases via mutations that render straightening of TM4 unfavorable, while maintaining the Ca^2+^-dependent rearrangement of TM6 (Figure 8A,B) (Dang et al., 2017; Paulino et al., 2017a). Indeed, in the Ca^2+^-free afTMEM16 scramblase, the pathway and cytosolic domain adopt conformations similar to those seen in the TMEM16A channel (Figure 8—figure supplement 1). Mutations that convert TMEM16A into a scramblase (Yu et al., 2015; Jiang et al., 2017) might re-enable the TM4 transition.

**Figure 8—figure supplement 1.**
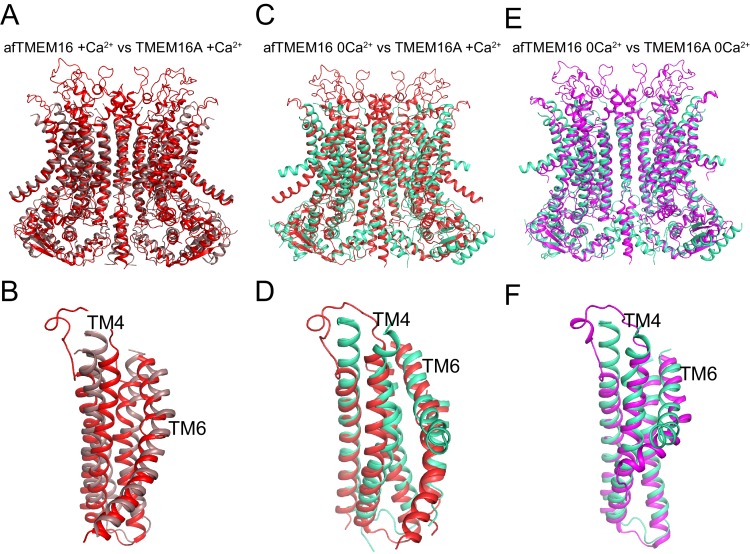
Structural comparison of Ca^2+^-bound and Ca^2+^-free afTMEM16 and TMEM16A. (**A–B**) Alignment of Ca^2+^-bound afTMEM16 (maroon) and Ca^2+^-bound TMEM16A (red, PDBID: 5OYB). A close-up view of the pathway is shown in (**B**) to highlight the closure of the permeation pathway in the TMEM16A channel by a rearrangement of TM4. (**C–D**) Alignment of Ca^2+^-free afTMEM16 (cyan) and Ca^2+^-bound TMEM16A (red). A close-up view of the pathway is shown in (**D**) to highlight how the TM4 helices adopt similar conformations. (**E–F**) Alignment of Ca^2+^-free afTMEM16 (cyan) and Ca^2+^-free TMEM16A (magenta, PDBID 5OYG). A close-up view of the pathway is shown in (**F**) to highlight how the TM4 helices adopt similar conformations and the TM6 bend at the same points. Note the different orientations of the intracellular portions of the TM6 in the two structures in the absence of Ca^2+^.

**Figure 8—figure supplement 2.**
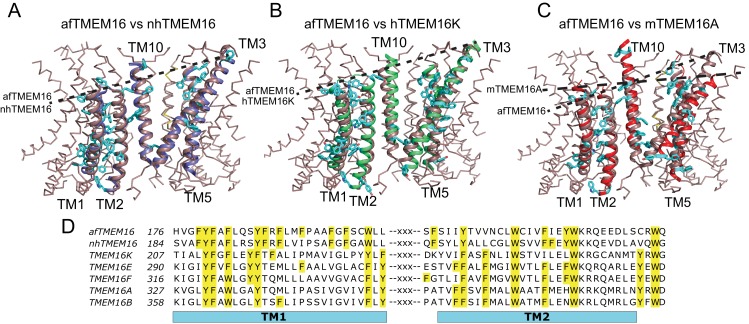
Architecture of the dimer cavity of TMEM16 proteins. (**A–C**) Structural alignment of the dimer cavities of nhTMEM16 (purple) (**A**), hTMEM16K (green) (**B**), or mTMEM16A (red) (**C**) to afTMEM16 (maroon). The transmembrane region of afTMEM16 is shown as ribbon. The five cavity-lining helices (TM1, TM2, and TM10 from one monomer, TM3 and TM5 from the other) from both displayed structures are shown in cartoon representation. Aromatic side chains from nhTMEM16 (**A**), hTMEM16K (**B**), and mTMEM16A (**C**) lining the cavity are shown as cyan sticks. The TM1 comprises 29 total residues, of which 11 are aromatic in afTMEM16, 10 in nhTMEM16, 8 in TMEM16K and 7 in TMEM16A. (**D**) Conservation of the aromatic residues in TM1 and TM2 within the TMEM16 family. Sequence alignment was generated using PROMALS3D (Pei and Grishin, 2014). Aromatic residues in TM1 and TM2 are highlighted in yellow.

**Figure 8—figure supplement 3.**
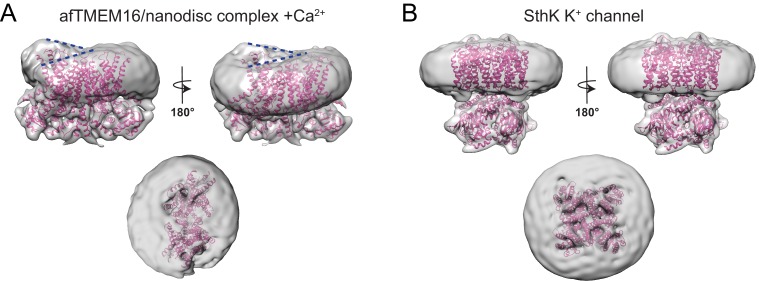
Effects of afTMEM16 on the nanodsic membrane are not observed in other unrelated membrane proteins. (**A–B**) The unmasked maps of the afTMEM16/nanodisc complex in the presence of 0.5 mM Ca^2+^ (**A**) and of the apo SthK potassium channel/nanodisc complex (Rheinberger et al., 2018) are shown low-pass filtered to 10 Å and at σ = 3.5. The respective atomic models are shown inside in pink (0.5 mM Ca^2+^, A; SthK apo PDBID 6CJQ, (**B**). Top panels: front and back (rotated 180^o^) views; Middle: top views; Dashed blue lines in the top panels in (**A**) trace the membrane bending near the dimer cavities. Note how similar bending is observed in the front and back views.

Our structures of afTMEM16 scramblase/nanodisc complexes suggest that lipid scrambling is enabled by two features of the TMEM16 architecture: the dimer cavities and the lipid pathway (Figures 5 and 7). The helices lining the dimer cavity are of different heights; TM3 and TM5 of one monomer are longer than the TM1 and TM2 from the other (Figure 5E) and the upper leaflet of the membrane bends to track this sloping. The shortest helices (TM1 and TM2) are unusually rich in aromatic side chains, which anchor TM segments to membrane/solution interfaces (O'Connell et al., 1990), creating a favorable environment for both phospholipid headgroups and tails (Figure 5E, Figure 8—figure supplement 2). This architecture is conserved in the nhTMEM16 and hTMEM16K scramblases (Figure 8—figure supplement 2A,B). In contrast, in the mTMEM16A channel TM1 is longer and contains fewer aromatics, and TM3 is shorter, such that the extracellular termini of these helices sit at comparable heights within the membrane (Figure 8—figure supplement 2C,D). The lesser degree of slanting in the dimer cavity-lining helices in mTMEM16A compared to afTMEM16 is consistent with the lack of membrane bending upon reconstitution of the mTMEM16A channel in nanodiscs (Dang et al., 2017). The observed bending is similar in the Ca^2+^-bound and Ca^2+^-free structures presented here (Figure 5), consistent with the idea that the dimer cavity does not undergo Ca^2+^-dependent rearrangements. In the nhTMEM16 and mTMEM16A structures, this cavity was proposed to be packed with lipids (Brunner et al., 2014; Dang et al., 2017). Indeed, while we could not identify well-defined lipids in this cavity, several densities attributable to partial acyl chains are visible between the extracellular termini of TM10 and TM2 as well as between TM3 and TM5, consistent with our hypothesis of membrane thickening around these helices (Figure 7—figure supplement 1). It is worth noting that reconstitution of unrelated non-scramblase membrane proteins into nanodisc, such as the SthK K^+^ channel (Rheinberger et al., 2018) (Figure 8—figure supplement 3), do not cause distortions in the nanodisc membrane, reinforcing the idea that these effects are specific to the afTMEM16 scramblase.

The Ca^2+^-dependent conformational rearrangements undergone by the pathway-lining helices correlate with changes in the density of the membrane near this conduit. In the Ca^2+^-bound conformation, the pathway is open and the density between TM4 and TM6 is weaker than in the rest of the nanodisc (Figure 5). We propose that this weakening reflects a thinning of the membrane in this region, as the hydrophilic residues lining the open pathway provide an energetically unfavorable environment for the acyl tails. This would cause the lipids to rearrange, to allow their headgroups to interact with the polar pathway and locally thin the membrane. Indeed, similar thinning and rearrangements in lipid orientation have been proposed based on MD simulations and on the ability of afTMEM16 to scramble lipids conjugated to PEG molecules with diameters larger than the width of the lipid pathway (Bethel and Grabe, 2016; Jiang et al., 2017; Lee et al., 2018; Malvezzi et al., 2018). In the Ca^2+^-free conformation, TM4 and TM6 rearrange to close the pathway preventing lipid access (Figure 3). The membrane exposed surface of the closed pathway is hydrophobic preventing thinning. Our finding that in the presence of the Cer24:0 lipid, the pathways of afTMEM16 are open while scrambling is inhibited suggests that its activity is not only regulated by Ca^2+^-dependent gating of the protein, but that other factors, such as the physico-chemical properties of the membrane, are also critical determinants of lipid scrambling. We propose that modulation of bilayer properties and composition might constitute secondary layers of regulatory control for the in vivo activation of scramblases.

Based on these observations, we propose a mechanism for lipid scrambling where the dimer cavities ‘prime’ the membrane by bending the outer membrane leaflet in opposite directions at the two sides of an open lipid pathway. This creates a membrane region that is highly curved, thin and disordered, all of which will facilitate lipid transfer between leaflets through the conduit formed by the open hydrophilic pathway (Figure 8E) (Bennett et al., 2009; Bruckner et al., 2009; Sapay et al., 2009). In the Ca^2+^-free conformation of the scramblase, the closed pathway prevents lipid entry and membrane thinning (Figure 8F). Similar mechanisms, where hydrophobic mismatches induce local distortion of membranes to lower the energy barrier for lipid movement through hydrophilic grooves, could be generally applicable to other scramblases.

## Materials and methods

**Key resources table:** 

Reagent type (species) or resource	Designation	Source or reference	Identifiers	Additional information
Biological sample (*Saccharomyces* *cerevisiae*)	FYG217 -URA	doi: 10.1038/nprot.2008.44		cells for expression, URA3 deletion
Biological sample (*Escherichia coli*)	BL21(DE3)	Stratagene		Cells for MSP1E3 expression
Recombinant DNA reagent (synthetic gene)	MSP1E3	Addgene https://www.addgene.org/20064/		
Recombinant DNA reagent (synthetic gene)	pET 28a	Addgene https://www.addgene.org/20064/		
Recombinant DNA reagent	pDDGFP2 (expression vector)	doi: 10.1038/nprot.2008.44	-	
Recombinant DNA reagent (*Aspergillus* *fumigatus*)	afTMEM16	doi:10.1038/ncomms3367	Gene ID: 3504033	
Software	Ana	M. Pusch, Istituto di Biofisica, Genova, Italy		http://users.ge.ibf.cnr.it/pusch/programs-mik.htm
Software	SigmaPlot		RRID:SCR_003210	
Software	Leginon	doi: 10.1016/j.jsb.2005.03.010		
Software	Relion	doi: 10.7554/eLife.18722		
Software	MotionCorr2	doi: 10.1038/nmeth.4193		
Software	CTFFIND4	doi: 10.1016/j.jsb.2015.08.008		
Software	cryoSPARC	doi: 10.1038/nmeth.4169		
Software	cisTEM	doi: 10.7554/eLife.35383		
Software	Bsoft (Blocres)	doi: 10.1016/j.jsb.2006.06.006		
Software	UCSF chimera	doi: 10.1002/jcc.20084		
Software	pymol	Schrödinger		

### Protein expression and purification

afTMEM16 was expressed and purified as described (Malvezzi et al., 2013). Briefly, *S. cerevisiae* carrying pDDGFP2 (Drew et al., 2008) with afTMEM16 were grown in yeast synthetic drop-out medium supplemented with Uracil (CSM-URA; MP Biomedicals) and expression was induced with 2% (w/v) galactose at 30° for 22 hr. Cells were collected, snap frozen in liquid nitrogen, lysed by cryomilling (Retsch model MM400) in liquid nitrogen (3 × 3 min, 25 Hz), and resuspended in buffer A (150 mM KCl, 10% (w/v) glycerol, 50 mM Tris-HCl, pH8) supplemented with 1 mM EDTA, 5 μg ml^−1^ leupeptin, 2 μg ml^−1^ pepstatin, 100 μM phenylmethane sulphonylfluoride and protease inhibitor cocktail tablets (Roche). Protein was extracted using 1% (w/v) digitonin (EMD biosciences) at 4°C for 2 hr and the lysate was cleared by centrifugation at 40,000 g for 45 min. The supernatant was supplemented with 1 mM MgCl_2_ and 10 mM Imidazole, loaded onto a column of Ni-NTA agarose resin (Qiagen), washed with buffer A + 30 mM Imidazole and 0.12% digitonin, and eluted with buffer A + 300 mM Imidazole and 0.12% digitonin. The elution was treated with Tobacco Etch Virus protease overnight to remove the His tag and then further purified on a Superdex 200 10/300 GL column equilibrated with buffer A supplemented with 0.12% digitonin (GE Lifesciences). The afTMEM16 protein peak was collected and concentrated using a 50 K_d_molecular weight cut off concentrator (Amicon Ultra, Millipore).

### Liposome reconstitution and lipid scrambling assay

Liposomes were prepared as described (Malvezzi et al., 2013), briefly lipids in chloroform (Avanti), including 0.4% w/w tail labeled NBD-PE, were dried under N_2_, washed with pentane and resuspended at 20 mg ml^−1^ in buffer B (150 mM KCl, 50 mM HEPES pH 7.4) with 35 mM 3-[(3-cholamidopropyl)dimethylammonio]−1- propanesulfonate (CHAPS). afTMEM16 was added at 5 µg protein/mg lipids and detergent was removed using four changes of 150 mg ml^−1^ Bio-Beads SM-2 (Bio-Rad) with rotation at 4°C. Calcium or EGTA were introduced using sonicate, freeze, and thaw cycles. Liposomes were extruded through a 400 nm membrane and 20 µl were added to a final volume of 2 mL of buffer B + 0.5 mM Ca(NO_3_)_2_ or 2 mM EGTA. The fluorescence intensity of the NBD (excitation-470 nm emission-530 nm) was monitored over time with mixing in a PTI spectrophotometer and after 100 s sodium dithionite was added at a final concentration of 40 mM. Data was collected using the FelixGX 4.1.0 software at a sampling rate of 3 Hz. All experiments with added ceramide lipids were carried out in the background of 1-palmitoyl-2-oleoyl-sn-glycero-3-phosphoethanolamine (POPE), 1-palmitoyl-2-oleoyl-sn-glycero-3-phospho-(1'-rac-glycerol) (POPG) at a ratio of 3:1. Ceramides tested include: N-stearoyl-D-erythro-sphingosine (Cer18:0), N-behenoyl-D-erythro-sphingosine (Cer22:0), N-lignoceroyl-D-erythro-sphinganine (Cer24:0), and N-nervonoyl-D-erythro-sphingosine (Cer24:1) all of which were tested at 1 mole% and 5 mole%. Chain length experiments were done in the background of 7PC:3 PG due to the availability of the long chain lipids. Lipids used include 1-palmitoyl-2-oleoyl-glycero-3-phosphocholine (POPC, 16:0-18:1C), POPG (16:0-18:1C), 1,2-dioleoyl-sn-glycero-3-phosphocholine (DOPC, 18:1C), 1,2-dioleoyl-sn-glycero-3-phospho-(1'-rac-glycerol) (DOPG, 18:1C), 1,2-dierucoyl-sn-glycero-3-phosphocholine (DEPC, 22:1C) and 1,2- dierucoyl-phosphatidylglycerol (DEPG, 22:1C).

### Quantification of scrambling activity

Quantification of the scrambling rate constants by afTMEM16 was determined as recently described (Lee et al., 2018; Malvezzi et al., 2018). Briefly, the fluorescence time course was fit to the following equation

(1) $$F_{tot}\left(t\right)=f_{0}\left(L_{i}^{PF}+(1-L_{i}^{PF})e^{-\gamma t}\right)+\frac{\left(1-f_{0}\right)}{D(\alpha +\beta )}\left\{\alpha \left(\lambda_{2}+\gamma \right)\left(\lambda_{1}+\alpha +\beta \right)e^{\lambda_{1}t}+\lambda_{1}\beta \left(\lambda_{2}+\alpha +\beta +\gamma \right)e^{\lambda_{2}t}\right\}$$

where F_tot_(t) is the total fluorescence at time t, L_i_^PF^ is the fraction of NBD-labeled lipids in the inner leaflet of protein free liposomes, γ=γ’[D] where γ’ is the second order rate constant of dithionite reduction, [D] is the dithionite concentration, f_0_ is the fraction of protein-free liposomes in the sample, α and β are the forward and backward scrambling rate constants, respectively, and

(2) $$\lambda_{1}=-\frac{\left(\alpha +\beta +\gamma \right)-\sqrt{{\left(\alpha +\beta +\gamma \right)}^{2}-4\alpha \gamma }}{2}\;\lambda_{2}=-\frac{\left(\alpha +\beta +\gamma \right)+\sqrt{{\left(\alpha +\beta +\gamma \right)}^{2}-4\alpha \gamma }}{2}$$

$$D=\left(\lambda_{1}+\alpha \right)\left(\lambda_{2}+\beta +\gamma \right)-\alpha \beta$$

The free parameters of the fit are f_0_, α and β while L_i_^PF^ and γ are experimentally determined from experiments on protein-free liposomes. In protein-free vesicles a very slow fluorescence decay is visible likely reflecting a slow leakage of dithionite into the vesicles or the spontaneous flipping of the NBD-labeled lipids. A linear fit was used to estimate the rate of this process was estimated to be L=(5.4 ± 1.6).10^−5^ s^−1^ (n > 160). For WT afTMEM16 and most mutants the leak is >2 orders of magnitude smaller than the rate constant of protein-mediated scrambling and therefore is negligible. All conditions were tested side by side with a control preparation in standard conditions. In some rare cases, this control sample behaved anomalously, judged by scrambling fit parameters outside three times the standard deviation of the mean for the WT. In these cases, the whole batch of experiments was disregarded.

### MSP1E3 purification and nanodisc reconstitution

MSP1E3 was expressed and purified as described (Ritchie et al., 2009). Briefly, MSP1E3 in a pET vector (Addgene #20064) was transformed into the BL21-Gold (DE3) strain (Stratagene). Transformed cells were grown in LB media supplemented with Kanamycin (50 mg l^−1^) to an OD_600_ of 0.8 and expression was induced with 1 mM IPTG for 3 hr. Cells were harvested and resuspended in buffer C (40 mM Tris-HCl pH 78.0, 300 mM NaCl) supplemented with 1% Triton X-100, 5 μg ml^−1^ leupeptin, 2 μg ml^−1^ pepstatin, 100 μM phenylmethane sulphonylfluoride and protease inhibitor cocktail tablets (Roche). Cells were lysed by sonication and the lysate was cleared by centrifugation at 30,000 g for 45 min at 4° C. The lysate was incubated with Ni-NTA agarose resin for 1 hr at 4°C followed by sequential washes with: buffer C + 1% triton-100, buffer C + 50 mM sodium cholate +20 mM imidazole and buffer C + 50 mM imidazole. The protein was eluted with buffer C + 400 mM imidazole, desalted using a PD-10 desalting column (GE life science) equilibrated with buffer D (150 mM KCl, 50 mM Tris pH 8.0) supplemented with 0.5 mM EDTA. The final protein was concentrated to ~8 mg ml^−1^ (~250 μM) using a 30 kDa molecular weight cut off concentrator (Amicon Ultra, Millipore), flash frozen and stored at −80°C.

Reconstitution of afTMEM16 in nanodiscs was carried out as follows, 3POPE:1POPG lipids in chloroform (Avanti) were dried under N_2_, washed with pentane and resuspended in buffer D and 40 mM sodium cholate (Anatrace) at a final concentration of 20 mM. Molar ratios of 1:0.8:60 MSP1E3:afTMEM16:lipids were mixed at a final lipid concentration of 7 mM and incubated at room temperature for 20 min. Detergent was removed via incubation with Bio-Beads SM-2 (Bio-Rad) at room temperature with agitation for 2 hr and then overnight with fresh Bio-Beads SM2 at a concentration of 200 mg ml^−1^. The reconstitution mixture was purified using a Superose6 Increase 10/300 GL column (GE Lifesciences) pre-equilibrated with buffer D plus 5 mM EDTA or 0.5 mM CaCl_2_ and the peak corresponding to afTMEM16-containing nanodiscs was collected for cryo electron microscopy analysis.

### Electron microscopy data collection

3.5 μL of afTMEM16-containing nanodiscs (7 mg mL^−1^) supplemented with 3 mM Fos-Choline-8-Fluorinated (Anatrace) was applied to a glow-discharged ﻿UltrAuFoil R1.2/1.3 300-mesh gold grid (Quantifoil) and incubated for one minute under 100% humidity at 15°C. Following incubation, grids were blotted for 2 s and plunge frozen in liquid ethane using a Vitrobot Mark IV (FEI). For the +C24:0 Ceramide/+Ca^2+^and EDTA samples as well as +Ca^2+^Dataset D (Supplementary file 1) micrographs were acquired on a Titan Krios microscope (FEI) operated at 300 kV with a K2 Summit direct electron detector (Gatan), using a slid width of 20 eV on a GIF Quantum energy filter and a Cs corrector with a calibrated pixel size of 1.0961 Å/pixel. A total dose of 62.61 e^–^/Å^2^ distributed over 45 frames (1.39 e^–^/ Å^2^/frame) was used with an exposure time of 9 s (200 ms/frame) and defocus range of −1.5 μm to −2.5 μm. For the +Ca^2+^datasets B and C (Supplementary file 1), micrographs were acquired on a Titan Krios microscope (FEI) operated at 300 kV with a K2 Summit direct electron detector with a calibrated pixel size of 1.07325 Å/pixel. A total dose of 69.97 e^–^/Å^2^ distributed over 50 frames (1.39 e^–^/ Å^2^/frame) was used with an exposure time of 10 s (200 ms/frame) and a defocus range of −1.5 μm to −2.5 μm. For all samples, automated data collection was carried out using Leginon (Suloway et al., 2005).

### Image processing

For all datasets except dataset D (in 0.5 mM Ca^2+^, Supplementary file 1), motion correction was performed using MotionCorr2 (Zheng et al., 2017) and contrast transfer function (CTF) estimation was performed using CTFFIND4 (Rohou and Grigorieff, 2015) both via Relion 2.0.3 (Kimanius et al., 2016). After manually picking ~2000 particles, the resulting 2D class-averages were used as templates for automated particle picking in Relion. The particles were extracted using a box size of 275 Å with 2xbinning and subjected to 2D classification ignoring CTFs until the first peak. For the Ca^2+^-free and ceramide data sets, particles selected from 2D classification (245,835 from Ca^2+^-free, and 185,451 from ceramide) were subjected to 3D classification using the nhTMEM16 crystal structure low-pass filtered to 40 Å as an initial model. For the 0.5 mM CaCl_2_ sample, datasets B and C were used combined to make dataset A; particles selected from the first round of 2D classification from each dataset were combined and subjected to a second round of 2D classification and the resulting 302,092 particles were subjected to the same 3D classification procedure. Particles from 3D classes with defined structural features (100,268 CaCl_2_, 70,535 Ca^2+^-free, 90,709 + C24:0 ceramide,) were combined and re-extracted without binning and refined without enforcing symmetry using an initial model generated in CryoSPARC (Punjani et al., 2017).

Initial refinement of the afTMEM16 dataset in 0.5 mM CaCl_2_ (Dataset B, C, which processed together form dataset A; Supplementary file 1) resulted in a map with a resolution of ~7 Å. The protein was symmetric, with the exception of the resolved portion of TM6 in each monomer (Figure 1—figure supplement 2). The complex was, however, not two-fold symmetric due to the off-center placement of the protein within the nanodisc (Figure 1—figure supplement 2). Therefore, data processing was carried out in parallel with C2 symmetry and without enforcing symmetry (in C1 symmetry). The particles from first C1 refinement were subjected to an additional round of 3D classification, using a mask that excluded the nanodisc and maintaining the particle orientations determined by the previous refinement. The best class from 3D classification with 27,948 particles was selected for further refinement and particle polishing. Masked refinement following particle polishing resulted in a 4.36 Å final map. To refine with C2 symmetry, the particles were polished and the nanodisc was removed using signal subtraction in Relion and the subtracted particles were refined using C2 symmetry, resulting in a 4.5 Å map. Using this map, a similar procedure to the C1 processing was carried out in which the best two classes from 3D classification without alignment applying a mask including the protein (37,146 particles) were selected for further refinement. Masked refinement of these classes yielded a 4.05 Å final density map. The C1 and C2 density maps were extensively compared and determined to be nearly identical except for the resolved portion of TM6 (Figure 1—figure supplement 2). The C2 map was used for model building while the C1 map was used for analysis of the afTMEM16/nanodisc complex.

For the Ca^2+^-free data set (referred to as ‘0 Ca^2+^”), the first refinement resulted in a map with a resolution of ~6 Å. As with the +Ca^2+^ sample, the protein was two-fold symmetric with the exception of the resolved portion of TM6 and the overall afTMEM16/nanodisc complex was not symmetric (Figure 1—figure supplement 2). Therefore, data was processed in parallel using both C1 and C2 symmetries as described above. The C1 map was classified and the best class from 3D classification with a mask excluding the nanodisc (38,550 particles) was selected for further refinement and particle polishing. Masked refinement following particle polishing resulted in a 4.00 Å final density map. Masked, C2 refinement following polishing and signal subtraction resulted in a 3.89 Å map. The C2 map was used for model building while the C1 map was used for analysis of the afTMEM16/nanodisc complex in 0 Ca^2+^.

For the data set in the presence of 0.5 mM CaCl_2_ and 5 mole% C24:0 Ceramide the selected particles were refined without symmetry, which resulted in a 4.2 Å resolution map. These particles were further classified in 3D with applied mask excluding the nanodiscs and maintaining angular information from the previous 3D refinement, from which two classes with 45,021 particles were selected for masked refinement which generated a final map of 3.74 Å. The resulting refinement showed that the nanodisc and the protein were C2 symmetric, therefore, further processing was completed with C2 symmetry enforced. Refinement resulted in a map with a resolution of 3.89 Å. An additional round of 3D classification was carried out using a mask excluding the nanodisc and maintaining the particle orientations determined by the previous refinement. The highest resolution class with 24,602 particles was selected for further refinement and particle polishing. Masked refinement following particle polishing resulted in a final map with a final average resolution of 3.59 Å.

The final resolution of all maps was determined by applying a soft mask around the protein and the gold-standard Fourier shell correlation (FSC) = 0.143 criterion using Relion Post Processing (Figure 1—figure supplement 1G). BlocRes from the Bsoft program was used to estimate the local resolution for all final maps (Heymann, 2001; Cardone et al., 2013)(Figure 1—figure supplement 1F).

The two other +Ca^2+^datasets (B and D, Figure 5—figure supplement 2, Supplementary file 1) mentioned were processed as described above up to the first refinement step due to the limited resolution (Figure 5—figure supplement 2). For processing +Ca^2+^dataset D in cryoSPARC, particles were picked using Difference of Gaussian picking (Voss et al., 2009) implemented through the appion program suite (Lander et al., 2009). For analysis of the final +Ca^2+^ dataset (A) in cyroSPARC, the particles originally picked in Relion were used. Extracted particles were classified in 2D and classes with structural features consistent with afTMEM16-nanodisc complexes were selected for ab initio model generation and classification. For each dataset, the best model and the associated particles were selected for homogeneous refinement followed by b-factor sharpening. Only the C24:0 dataset had a resolution better than 6 Å from cryoSPARC so these models were not used for analysis other than to confirm the observed membrane bending and thinning (Supplementary file 1, Figure 5—figure supplement 3). For processing in cisTEM, particles from 3D classes selected in Relion were used to create an mrc stack and imported. Several rounds of automatic refinement and manual refinement with both local and global searches were carried out. Using auto-masking and manual refinement the final resolution for the +Ca^2+^dataset was~5 Å and was therefore not used for an analysis other than to confirm the observed effects on the membrane (Supplementary file 1, Figure 5—figure supplement 3).

### Model building, refinement, and validation

The maps were of high quality and we built atomic models for each structure for the transmembrane domain, most of the cytosolic region and connecting loops (Figure 1—figure supplement 4), as well as identifying seven partial lipids in the +C24:0 Ceramide structure (Figure 7—figure supplement 1). The model of afTMEM16 in the presence of ceramide was built first, due to the higher resolution of the map. The crystal structure of nhTMEM16 (PDBID 4wis) was used as a starting model and docked into the density map using chimera and the jiggle fit script in COOT (Emsley et al., 2010) (https://www2.mrc-lmb.cam.ac.uk/groups/murshudov/index.html), and mutated to match the afTMEM16 sequence. The final model contains residues 13–54, 60–119, 126–259, 270–312, 316–399, 418–461, 490–597, 602–657 and 705–724 and the following residues were truncated due to missing side chain density: L43, Q49, R53, K69, K70, E94, K100, Q102, K129, H130, D137, K257, E258, L316, E461, H555, F705, K713, E714, and E717. The model was improved iteratively by real space refinement in PHENIX imposing crystallographic symmetry and secondary structure restraints followed by manual inspection and removal of outliers. The model for the +Ca^2+^structure was built using the refined +C24:0 Ceramide structure (PDBID 6E1O) as an initial model, that was docked into the +Ca^2+^density using the jigglefit script in COOT (Emsley et al., 2010) (https://www2.mrc-lmb.cam.ac.uk/groups/murshudov/index.html), manually inspected, and refined using real space refinement in PHENIX as above (Adams et al., 2010; Afonine et al., 2013). The final model contains residues 13–54, 60–119, 128–258, 270–312, 316–400, 420–460, 490–594, 602–659 and 705–724, and the following residues were truncated due to missing side chain density: D17, Q49, R53, K100, Q102, E104, K129, H130, N135, D137, E164, H247, K254, K257, H460, K550, H595, R710, K713, E714, E717, and L720. The model for the 0 Ca^2+^ structure was built using the +Cera model as a starting point and regions where the density differed were built manually (TM3, TM4 and TM6) or rigid body refined where smaller rearrangements were observed. The final model contains residues 13–54, 60–119, 128–258, 270–312, 316–400, 420–460, 490–594, 602–659 and 705–724, and the following residues were truncated due to missing side chain density: D17, F36, R53, K70, E94, K100, Q102, E132, K241, H247, R250, K257, E310, K317, K634, K642, Y654, R704, R708, E714, E717. For all three structures, the density in the regions of TM3 and TM4 and the connecting loop (residues 274–352) was less well-defined than remainder of the structure and the model may be less accurate in this area.

To validate the refinement, the FSC between the refined model and the final map was calculated (FSCsum) (Figure 1—figure supplement 1H). To evaluate for over-fitting, random shifts of up to 0.3 Å were introduced in the final model and the modified model was refined using PHENIX against one of the two unfiltered half maps. The FSC between this modified-refined model and the half map used in refinement (FSCwork) was determined and compared to the FSC between the modified-refined model and the other half map (FSCfree) which was not used in validation or refinement. The similarity in these curves indicates that the model was not over-fit (Figure 1—figure supplement 1H). The quality of all three models was assessed using MolProbity (Chen et al., 2010) and EMRinger (Barad et al., 2015), both of which indicate that the models are of high quality (Figure 1—figure supplement 1H).

### Difference map calculation

To compare the maps resulting from C1 and C2 processing in the 0 Ca^2+^ and +Ca^2+^ structures, we calculated a difference map between the two volumes using the volume operation subtract function in chimera (Pettersen et al., 2004) (Figure 1—figure supplement 2). We used ‘omit density’ to assign the placement of several lipids in the 0 Ca^2+^ and +C24:0 Ceramide structures and the Ca^2+^ ions in the +Ca^2+^and + Cera structures which were calculated using the ‘phenix.real_space_diff_map’ function in PHENIX (Adams et al., 2010; Afonine et al., 2013; Dang et al., 2017). Briefly, completed models without the ligand in question were used to generate a theoretical density map which was subtracted from the experimental density map. In the subtracted map, areas of ligand density appeared as positive density.

### Gramicidin fluorescence assay

The gramicidin channel-based fluorescence assay for monitoring changes in lipid bilayer properties was implemented using large unilamellar vesicles (LUVs) incorporating gramicidin (gA) and loaded with 8-aminonaphthalene-1,3,6-trisulfonic acid, disodium salt (ANTS), following published protocols (Ingólfsson and Andersen, 2010; Ingólfsson et al., 2011). Vesicles composed of 1-palmitoyl-2-oleoyl-sn-glycero-3-phosphoethanolamine (POPE) and 1-palmitoyl-2-oleoyl-sn-glycero-3-phospho-1'-rac-glycerol (POPG) in 3:1 (mol/mol) proportion in chloroform were mixed with a gA to lipid molar ratio of 1:40,000. An additional 0, 1, or 5 mole% of C24:0 ceramide (d18:1/24:0) or C24:1 ceramide (d18:1/24:1) in chloroform were added to the gA-phospholipid mix. The lipid mixtures were dried under nitrogen to remove chloroform and further dried in vacuum overnight. Lipids were rehydrated with fluorophore-containing buffer solution (25 mM ANTS, 100 mM NaNO_3_, and 10 mM HEPES), vortexed for 1 min, and incubated at room temperature for at least 3 hr while shielded from light. The solutions were sonicated for 1 min at low power and subjected to six freeze-thaw cycles. The samples then were extruded 21 times with an Avanti Polar Lipids mini-extruder (Avanti) and 0.1 mm polycarbonate filter. The samples were extruded at 35–40°C. Extravesicular ANTS was removed with PD-10 desalting column (GE Healthcare). The average LUV diameter was ~130 nm, with an average polydispersity index (PDI) of 10%, as determined using dynamic light scattering. LUV stock solutions were stored at room temperature with an average shelf life of 7 days.

The time course of the ANTS fluorescence was measured at 25^o^C with an SX-20 stopped-flow spectrofluorometer (Applied Photophysics), with an LED light source. Excitation was at 352 nm and emission was recorded above 450 nm with a high-pass filter; the deadtime of the instrument is ≈ 2 ms with a sampling rate of 5000 points/s. Samples were prepared by diluting the stock lipid concentration with buffer solution (140 mM NaOH and 10 mM HEPES) to 125 μM LUV; each sample was incubated for at least 10 min before several 1 s mixing reactions. Each sample was first mixed with the control buffer (no Tl^+^), followed by mixing with the quench solution (50 mM TINO_3_94 mM NaNO_3_ and 10 mM HEPES). Experiments are conducted with two independently prepared populations of vesicles per lipid/ceramide combination and traces are analyzed in MATLAB (MathWorks).

The fluorescence quench rate therefore was determined as described (Ingólfsson and Andersen, 2010) by fitting the time course to a stretched exponential function (Berberan-Santos et al., 2005):

(3) $$F\left(t\right)=F\left(\infty \right)+\left[F\left(0\right)-F\left(\infty \right)\right]\cdot \text{exp}\left[-{\left(t/\tau_{0}\right)}^{\beta }\right]$$

*F*(*t*) denotes the fluorescence intensities at time *t*; β (0 < β ≤1) is a parameter that accounts for the dispersity of the vesicle population; and τ_o_ is a parameter with dimension of time. *F*(0), *F*(∞), β and τ_o_ were determined from a nonlinear least squares fit of Eq. 1 to each individual fluorescence trace, and the quench rate was determined (Berberan-Santos et al., 2005):

(4) $$Rate(t)=\frac{\beta }{\tau 0}{\left[\frac{t}{\tau 0}\right]}^{\left(\beta -1\right)}$$

evaluated at *t* = 2 ms. The ceramide-induced changes in the quench rate then was evaluated as

$$Rate\text{/}Rate_{\text{cntrl}}\text{=}\frac{Rate{\left(t\right)}_{\text{ceramide }}}{Rate{\left(t\right)}_{\text{cntrl}}}$$

where the subscripts ‘ceramide’ and ‘cntrl’ denote the rates observed in the presence and absence of ceramide.

### Data availability

The three-dimensional cryo-EM density maps of the calcium-bound, calcium-free, and Ca^2+^-bound in the presence of C24:0 Ceramide afTMEM16/nanodisc complexes have been deposited in the Electron Microscopy Data Bank under accession numbers EMD-8948, EMD-8931, and EMDB-8959, respectively. The deposition includes corresponding masked and unmasked maps, the mask used for the final FSC calculation, and the FSC curves. Coordinates for the models of the calcium-bound, calcium-free, and Ca^2+^-bound ceramide inhibited states have been deposited in the Protein Data Bank under accession numbers 6E0H, 6DZ7, and 6E1O respectively. All other data are available from the corresponding author upon reasonable request.

## Additional files

Source data 1. Raw data of the representative fluorescence decay traces of afTMEM16-mediated lipid scrambling in liposomes formed from lipids with different chain length and saturation.

Source data 2. Raw data of the representative fluorescence decay traces of afTMEM16-mediated lipid scrambling in liposomes containing different Ceramide lipids.

Supplementary file 1. Summary of cryo-EM datasets utilized in this work. Detailed processing procedures are described in the methods. Note that +Ca^2+^dataset C was analyzed independently only up to 2D classification; after 2D classification it was combined with dataset B to generate dataset A, which yielded the final high-resolution +Ca^2+^ map.

Supplementary file 2. Average values of the scrambling rate constants of afTMEM16 in short (16–18C) and long (22:1) chain lipids. The following parameters were derived as described ^6^ by fitting the data to Eq. 1: f_0_ is the fraction of empty liposomes, α and β are the forward and reverse scrambling rate constants, γ is the reduction rate constant by dithionite, L_i_^PF^ is the fraction of NBD-labeled lipids in the inner leaflet of a protein-free vesicle, n is the number of independent experiments. Data is reported as the mean ±SD. * denotes values that were constrained during fitting.

Supplementary file 3. Statistics of cryo-EM data collection, 3D reconstruction and model refinement.

Supplementary file 4. Average values of the scrambling rate constants of afTMEM16 in the presence of various ceramides. The following parameters were derived by fitting the data to Eq. 1: f_0_ is the fraction of empty liposomes, α and β are the forward and backward scrambling rate constants, γ is the reduction rate constant by dithionite, L_i_^PF^ is the fraction of NBD-labeled lipids in the inner leaflet of a protein-free vesicle, n is the number of independent experiments. Data is reported as the mean ±SD.

### Data availability

Masked and unmasked cryoEM maps have been deposited in the EMDB database under accession codes: EMD-8931, EMD-8948, EMD-8959. Atomic coordinates have been deposited in the PDB database under accession codes: 6E0H, 6DZ7, 6E1O. Motion corrected EM micrographs are deposited in the EMPIAR database under accession codes: EMPIAR-10240, EMPIAR-10239,EMPIAR-10241. Key parameters of the fluorescence time courses are detailed in the tables and figures and representative traces used in the figures have been provided as source data files. The raw fluorescence time courses are available upon request to the corresponding author.

The following datasets were generated:

FalzoneMEAccardiA2018afTMEM16 reconstituted in nanodiscs in the presence of Ca2+Protein Data Bank6E0H

FalzoneMEAccardiA2018CryoEM data from Structural basis of Ca2+-dependent activation and lipid transport by a TMEM16 scramblaseProtein Data Bank6D2710.7554/eLife.43229PMC635519730648972

FalzoneMEAccardiA2018afTMEM16 reconstituted in nanodiscs in the presence of Ca2+ and ceramide 24:0Protein Data Bank6E1O

FalzoneMEAccardiA2018Ca2+-free afTMEM16 in nanodiscElectron Microscopy Data BankEMD-8931

FalzoneMEAccardiA2018Ca2+-bound afTMEM16 in nanodiscElectron Microscopy Data BankEMD-8948

FalzoneMEAccardiA2018Structure of afTMEM16 in nanodisc in the presence of 0.5 mM Ca2+ and 0.5 mol% Ceramide 24:0Electron Microscopy Data BankEMD-8959

MariaE FalzoneAlessioAccardi2019Motion Corrected micrographs of the afTMEM16/nanodisc complex in the absence of Ca2+EMPIAREMPIAR-10241

MariaE FalzoneAlessioAccardi2019Motion Corrected micrographs of the afTMEM16/nanodisc complex in the presence of 0.5 mM Ca2+EMPIAREMPIAR-10239

MariaE FalzoneAlessioAccardi2019Motion Corrected micrographs of the afTMEM16/nanodisc complex in the presence of 0.5 mM Ca2+ and 5 mol% Ceramide 24:0EMPIAREMPIAR-10240
