# Supplementary material for: Structural basis of Ca2+-dependent activation and lipid transport by a TMEM16 scramblase
Source: eLife. 2019 Jan 16;8:e43229. doi: 10.7554/eLife.43229 (PMC6355197; doi:10.7554/eLife.43229)
Supplement: Supplementary file 1. — Detailed processing procedures are described in the methods. Note that +Ca2+dataset C was analyzed independently only up to 2D classification; after 2D classification it was combined with dataset B to generate dataset A, which yielded the final high-resolution +Ca2+ map. [file elife-43229-supp1.docx]

| Condition | Dataset identifier | Particle Picking | Processing Program | Resolution (Å) |
| --- | --- | --- | --- | --- |
| +Ca^2+^ | D | DoG picker | cryoSPARC 1 | ~8 |
| +Ca^2+^ | B | Relion-template | Relion | 4.5 |
|  | B | Relion-template | cisTEM | ~4.5 |
| +Ca^2+^ | C | Relion-template | Relion | N/A |
| +Ca^2+^ | A (B+C) | Relion-template | Relion | 4 |
| +Ca^2+^ | A (B+C) | Relion-template | cryoSPARC 2 | ~7 |
| 0 Ca^2+^ |  | Relion-template | Relion | 3.9 |
| +C24:0 Ceramide/+Ca^2+^ |  | Relion-template | Relion | 3.6 |

**Supplementary Table 2 Summary of cryo-EM datasets utilized in this work**. Detailed processing procedures are described in the methods. Note that +Ca^2+^ dataset C was analyzed independently only up to 2D classification; after 2D classification it was combined with dataset B to generate dataset A, which yielded the final high resolution +Ca^2+^ map.
