# Supplementary material for: Structural basis of Ca2+-dependent activation and lipid transport by a TMEM16 scramblase
Source: eLife. 2019 Jan 16;8:e43229. doi: 10.7554/eLife.43229 (PMC6355197; doi:10.7554/eLife.43229)
Supplement: Supplementary file 2. — The following parameters were derived as described 6 by fitting the data to Eq. 1: f0 is the fraction of empty liposomes, α and β are the forward and reverse scrambling rate constants, γ is the reduction rate constant by dithionite, LiPF is the fraction of NBD-labeled lipids in the inner leaflet of a protein-free vesicle, n is the number of independent experiments. Data is reported as the mean ±SD. * denotes values that were constrained during fitting. [file elife-43229-supp2.docx]

| Lipid  (chain length) | Condition | f_0_ | α (s^-1^) | β (s^-1^) | γ (s^-1^) | *L_i_^PF^* | n |
| --- | --- | --- | --- | --- | --- | --- | --- |
| 3 POPE: 1 POPG  (16:0-18:1C) | Protein Free | n.a | n.a | n.a. | 0.05±0.02 | 0.49 ± 0.05 | 20 |
|  | afTMEM16 + Ca^2+^ | 0.27±0.13 | 0.099±0.047 | 0.055±0.040 | 0.052±0.016 | 0.50 ± 0.04 | 23 |
|  | afTMEM16 0 Ca^2+^ | 0.21±0.07 | (1.7± 1.1)∙10^-3^ | (5.0±2.0)∙10^-4^ | 0.036±0.014 | 0.49 ± 0.05 | 15 |
| 7 POPC: 3 POPG  (16:0-18:1C) | Protein Free | n.a | n.a | n.a. | 0.055±7.0∙10^-3^ | 0.49 ± 0.02 | 8 |
|  | afTMEM16 + Ca^2+^ | 0.29±0.03 | 0.063±0.017 | 0.054 ± 0.029 | 0.054±7∙10^-3^ | 0.49 ± 0.02 | 9 |
|  | afTMEM16 0 Ca^2+^ | 0.41±0.07 | (4.2± 1.6)∙10^-3^ | (1.9±0.9)∙10^-4^ | 0.05±0.02 | 0.55 ± 0.08 | 6 |
| 7 DOPC: 3 DOPG  (18:1-18:1C) | Protein Free | n.a | n.a | n.a. | 0.16±0.02 | 0.47±7.5∙10^-3^ | 12 |
|  | afTMEM16 + Ca^2+^ | 0.42±0.01 | 0.14±0.02 | 0.16±0.05 | 0.13 ± 0.03 | 0.47±7.5∙10^-3^ | 9 |
|  | afTMEM16 0 Ca^2+^ | 0.42±0.02 | (6.4 ±3.5)∙10^-3^ | (4.1±2.4)∙10^-3^ | 0.06 ± 8.5∙10^-3^ | 0.47±4.3∙10^-3^ | 9 |
| 7 DEPE: 3 DEPG  (22:1-22:1C) | Protein Free | n.a | n.a | n.a. | 0.071±0.004 | 0.371±8∙10^-3^ | 9 |
|  | afTMEM16 + Ca^2+^ | 0.21* | (2.7±0.3)∙10^-4^ | (1.6±0.2)∙10^-4^ | 0.042±0.003 | 0.371±6∙10^-3^ | 9 |
|  | afTMEM16 0 Ca^2+^ | 0.21* | (1.9±0.4)∙10^-4^ | (1.7±0.2)∙10^-4^ | 0.043±5∙10^-3^ | 0.371±6∙10^-3^ | 9 |

**Supplementary Table 3.** Average values of the scrambling rate constants of afTMEM16 in short (16-18C) and long (22:1) chain lipids. The following parameters were derived as described ^6^ by fitting the data to Eq. 1: f_0_ is the fraction of empty liposomes, α and β are the forward and reverse scrambling rate constants, γ is the reduction rate constant by dithionite, L_i_^PF^ is the fraction of NBD-labeled lipids in the inner leaflet of a protein-free vesicle, n is the number of independent experiments. Data is reported as the mean±StDev. * denotes values that were constrained during fitting.
