# Supplementary material for: Structural basis of Ca2+-dependent activation and lipid transport by a TMEM16 scramblase
Source: eLife. 2019 Jan 16;8:e43229. doi: 10.7554/eLife.43229 (PMC6355197; doi:10.7554/eLife.43229)
Supplement: Supplementary file 3. [file elife-43229-supp3.docx]

|  | **Ca^2+^** | | **Apo** | **Ceramide** |
| --- | --- | --- | --- | --- |
| **Data Collection and Processing** | |  |  |  |
| Microscope | FEI titan Krios | | FEI titan Krios | FEI titan Krios |
| Camera | Gatan K2 Summit | | Gatan K2 Summit + GIF | Gatan K2 Summit + GIF |
| Nominal Magnification | 22500x | | 105000x | 105000 |
| Voltage (kV) | 300 | | 300 | 300 |
| Exposure time frame/total (s) | 0.2/10 | | 0.2/9 | 0.2/9 |
| Number of Frames | 50 | | 45 | 45 |
| Electron exposure total (e^-^/Å^2^) | 69.97 | | 62.61 | 62.61 |
| Nominal Defocus range (µm) | -1.5 to -2.5 | | -1.5 to -2.5 | -1.5 to -2.5 |
| Pixel Size (Å) | 1.07325 | | 1.0961 | 1.0961 |
| Symmetry imposed | C2 | | C2 | C2 |
| Initial/Final micrographs (no.) | 5,524/2,383 | | 4,656/3,105 | 2,143/1,495 |
| Initial Particle Images (no.) | 1,034,678 | | 752,911 | 521,688 |
| Final Particle Images (no.) | 37,146 | | 70,536 | 24,602 |
| Map resolution (Å) (0.143 FSC) | 4.05 | | 3.89 | 3.59 |
| Map Resolution Range (Å) | 3.5-6 | | 3.2-5 | 3-5 |
| **Refinement** |  | |  |  |
| Initial model used (PDB code) | 6E1O | | 6E1O | 4WIS |
| Model Resolution (Å) (0.5 FSC) | 4.11 | | 3.96 | 3.65 |
| Map Sharpening B factor (Å^2^) | -159.94 | | -135.09 | -98.629 |
| **Model Composition** |  | |  |  |
| Non-hydrogen Atoms | 9491 | | 9284 | 9750 |
| Protein residues | 1392 | | 1364 | 1412 |
| Ligands | 4 | | 18 | 4 |
| **R.m.s. deviations** |  | |  |  |
| Bond length (A) | 0.009 | | 0.010 | 0.008 |
| Bond angles (°) | 1.42 | | 1.186 | 1.349 |
| **Validation** |  | |  |  |
| MolProbity score | 1.78 | | 1.74 | 1.68 |
| Clashscore | 4.98 | | 3.57 | 4.62 |
| Poor Rotomers (%) | 0 | | 0.2 | 0.88 |
| EMRinger score | 1.53 | | 1.93 | 2.30 |
| **Ramachandran Plot** |  | |  |  |
| Favored (%) | 91.20 | | 88.27 | 93.11 |
| Allowed (%) | 8.8 | | 11.73 | 6.89 |
| Disallowed (%) | 0 | | 0 | 0 |

**Supplementary Table 1.** Statistics of cryo-EM data collection, 3D reconstruction and model refinement.
