# Supplementary material for: Structural basis of Ca2+-dependent activation and lipid transport by a TMEM16 scramblase
Source: eLife. 2019 Jan 16;8:e43229. doi: 10.7554/eLife.43229 (PMC6355197; doi:10.7554/eLife.43229)
Supplement: Supplementary file 4. — The following parameters were derived by fitting the data to Eq. 1: f0 is the fraction of empty liposomes, α and β are the forward and backward scrambling rate constants, γ is the reduction rate constant by dithionite, LiPF is the fraction of NBD-labeled lipids in the inner leaflet of a protein-free vesicle, n is the number of independent experiments. Data is reported as the mean ±SD. [file elife-43229-supp4.docx]

| Ceramide | % | Condition | f_0_ | α (s^-1^) | β (s^-1^) | γ (s^-1^) | L_i_^PF^ | n |
| --- | --- | --- | --- | --- | --- | --- | --- | --- |
| 0 | n.a | Protein Free | n.a | n.a | n.a. | 0.05±0.02 | 0.49±0.05 | 20 |
|  |  | afTMEM16 + Ca^2+^ | 0.27±0.13 | 0.099±0.047 | 0.06 ± 0.04 | 0.05±0.02 | 0.50±0.04 | 23 |
|  |  | afTMEM16 0 Ca^2+^ | 0.21±0.07 | (1.7±1.1)∙10^-3^ | (5.0±2.0)∙10^-4^ | 0.04±0.01 | 0.49±0.05 | 15 |
| 18:0 | 1% | Protein Free | n.a | n.a | n.a. | 0.063±5∙10^-3^ | 0.48±0.03 | 4 |
|  |  | afTMEM16 + Ca^2+^ | 0.47±0.03 | 0.21±0.09 | 0.23±0.18 | 0.08 ± 0.01 | 0.49±0.03 | 5 |
|  |  | afTMEM16 0 Ca^2+^ | 0.34±0.15 | (8.74±3.41)∙10^-4^ | (4.6± 2.0) ∙10^-4^ | 0.05±0.02 | 0.41±0.2 | 4 |
|  | 5% | Protein Free | n.A | n.a | n.a. | 0.061±6∙10^-3^ | 0.48±0.01 | 4 |
|  |  | afTMEM16 + Ca^2+^ | 0.28±0.17 | 0.21±0.12 | 0.04±0.03 | 0.066±9∙10^-3^ | 0.47±0.01 | 4 |
|  |  | afTMEM16 0 Ca^2+^ | 0.36±0.19 | (9.7±3.6)∙10^-4^ | (5.0±2.4)∙10^-4^ | 0.059±6∙10^-3^ | 0.48±0.01 | 4 |
| 22:0 | 1% | Protein Free | n.a | n.a | n.a. | 0.067±6∙10^-3^ | 0.506±4∙10^-3^ | 9 |
|  |  | afTMEM16 + Ca^2+^ | 0.28±0.02 | 0.08±0.06 | 0.03±0.02 | 0.066±9∙10^-3^ | 0.506±3∙10^-3^ | 6 |
|  |  | afTMEM16 0 Ca^2+^ | 0.28±0.04 | (5.43±0.37)∙10^-4^ | (4.1±1.6)∙10^-4^ | 0.05±0.02 | 0.051±7∙10^-3^ | 6 |
|  | 5% | Protein Free | n.a | n.a | n.a. | 0.07 ± 0.02 | 0.43 ± 0.05 | 9 |
|  |  | afTMEM16 + Ca^2+^ | 0.18±0.02 | (3.78±1.82)∙10^-4^ | (2.1±1.5)∙10^-4^ | 0.06 ± 0.02 | 0.43 ± 0.02 | 8 |
|  |  | afTMEM16 0 Ca^2+^ | 0.17±0.02 | (1.61±0.43)∙10^-4^ | (1.4±0.6)∙10^-4^ | 0.06±0.02 | 0.43±0.05 | 8 |
| 24:0 | 1% | Protein Free | n.a | n.a | n.a. | 0.044±3∙10^-3^ | 0.45±0.03 | 6 |
|  |  | afTMEM16 + Ca^2+^ | 0.31±0.12 | 0.05±0.02 | 0.01±7∙10^-3^ | 0.042±3∙10^-3^ | 0.44±0.03 | 5 |
|  |  | afTMEM16 0 Ca^2+^ | 0.32±0.12 | (3.7±1.7)∙10^-4^ | (2.0±0.5)∙10^-4^ | 0.039±4∙10^-3^ | 0.44±0.02 | 5 |
|  | 5% | Protein Free | n.a | n.a | n.a. | 0.047±5∙10^-3^ | 0.33±0.02 | 4 |
|  |  | afTMEM16 + Ca^2+^ | 0.19±0.04 | (3.9±2.3)∙10^-4^ | (2.4±1.5)∙10^-4^ | 0.047 ± 7∙10^-3^ | 0.32 ± 0.01 | 6 |
|  |  | afTMEM16 0 Ca^2+^ | 0.19±0.04 | (4. 8±4.7)∙10^-4^ | (2±2)∙10^-4^ | 0.043±5∙10^-3^ | 0.32 ± 0.01 | 6 |
| 24:1 | 1% | Protein Free | n.a | n.a | n.a. | 0.044 ± 6∙10^-3^ | 0.50±0.02 | 9 |
|  |  | afTMEM16 + Ca^2+^ | 0.33±0.12 | 0.09 ± 0.02 | 0.055±0.031 | 0.05±0.01 | 0.50±0.02 | 9 |
|  |  | afTMEM16 0 Ca^2+^ | 0.32± 0.12 | (2.6±1.7)∙10^-4^ | (1.8±1.1)∙10^-4^ | 0.036±6∙10^-3^ | 0.50± 0.02 | 9 |
|  | 5% | Protein Free | n.a | n.a | n.a. | 0.050±8∙10^-3^ | 0.50±0.02 | 7 |
|  |  | afTMEM16 + Ca^2+^ | 0.27±0.06 | 0.064±0.037 | 0.03±0.04 | 0.057 ± 0.014 | 0.50±0.01 | 8 |
|  |  | afTMEM16 0 Ca^2+^ | 0.27±0.06 | (5.2±1.8)∙10^-4^ | (1.96±0.28)∙10^-4^ | 0.040 ± 7∙10^-3^ | 0.50±0.01 | 6 |

**Supplementary Table 4 Average values of the scrambling rate constants of afTMEM16 in the presence of various ceramides**. The following parameters were derived by fitting the data to Eq. 1: f_0_ is the fraction of empty liposomes, α and β are the forward and backwards scrambling rate constants, γ is the reduction rate constant by dithionite, L_i_^PF^ is the fraction of NBD-labeled lipids in the inner leaflet of a protein-free vesicle, n is the number of independent experiments. Data is reported as the mean±StDev.
